# Epistasis shapes the fitness landscape of an allosteric specificity switch

**DOI:** 10.1038/s41467-021-25826-7

**Published:** 2021-09-21

**Authors:** Kyle K. Nishikawa, Nicholas Hoppe, Robert Smith, Craig Bingman, Srivatsan Raman

**Affiliations:** 1 Department of Biochemistry, University of Wisconsin-Madison, Madison, WI USA; 2 Department of Bacteriology, University of Wisconsin-Madison, Madison, WI USA; 3 Department of Chemical and Biological Engineering, University of Wisconsin-Madison, Madison, WI USA

**Keywords:** X-ray crystallography, Transcription factors, Molecular evolution

## Abstract

Epistasis is a major determinant in the emergence of novel protein function. In allosteric proteins, direct interactions between inducer-binding mutations propagate through the allosteric network, manifesting as epistasis at the level of biological function. Elucidating this relationship between local interactions and their global effects is essential to understanding evolution of allosteric proteins. We integrate computational design, structural and biophysical analysis to characterize the emergence of novel inducer specificity in an allosteric transcription factor. Adaptive landscapes of different inducers of the designed mutant show that a few strong epistatic interactions constrain the number of viable sequence pathways, revealing ridges in the fitness landscape leading to new specificity. The structure of the designed mutant shows that a striking change in inducer orientation still retains allosteric function. Comparing biophysical and functional properties suggests a nonlinear relationship between inducer binding affinity and allostery. Our results highlight the functional and evolutionary complexity of allosteric proteins.

## Abstract (web summary)

Epistasis plays an important role in the evolution of novel protein functions because it determines the mutational path a protein takes. Here, the authors combine functional, structural and biophysical analyses to characterize epistasis in a computationally redesigned ligand-inducible allosteric transcription factor and found that epistasis creates distinct biophysical and biological functional landscapes.

## Introduction

Interactions between mutations direct the evolution of protein function^1^. As proteins evolve, they follow paths through the fitness landscape to reach a fitness peak that represents a novel function^2^. For N mutations required to confer novel function, there are N! possible pathways connecting the start and end states. However, some pathways may not be evolutionarily favorable due to epistasis—a phenomenon that occurs when the sequence background into which a mutation is introduced changes the functional effect of that mutation. The non-additivity due to epistasis strongly influences the sequence trajectory a protein takes to gain new function^1,3–5^. Therefore, understanding the nature of epistatic interactions is the foundation for investigating the mechanisms leading to novel protein function^6^.

Epistasis is generally categorized as specific or nonspecific based on cause-effect relationships between the interactions of mutations and their outcome. Specific epistasis occurs between a limited number of residues that typically physically interact, leading to nonadditive changes in thermodynamically driven biophysical properties such as protein stability or affinity^7^. Specific epistasis has been extensively investigated in protein-protein, protein-ligand, protein-DNA interactions^5,8–16^. Nonspecific epistasis occurs when mutations are nonadditive with respect to protein traits when combined^17–20^. Such mutations can be spatially distant such as a global suppressor that can interact with many destabilizing mutations with low pairing specificity^4,21,22^.

In this study, we examine the role of epistasis in the evolution of ligand specificity in an allosteric transcription factor. Allostery is a fundamental mechanism by which proteins recognize environmental cues (such as binding of an inducer or effector) within a localized region resulting in modulation of function at a distal site^23,24^. Mutations in the binding pocket that trigger the allosteric network have the potential to create new nonspecific epistatic interactions at the level of protein function, beyond the physical interactions commonly seen in specific epistasis. As allosteric proteins evolve toward new function, such as orthologs in different organisms, their inducer specificity changes to adapt to the new environment^25^. Allosteric proteins may accrue mutations during evolution that would simultaneously affect specificities for old and new inducers. Further, these mutations may also impact function by affecting the capability of the protein to produce an allosteric change in response to an inducer^26,27^. For an allosteric transcription factor (aTF), a function is the outcome of affinity for the inducer ligand, affinity for DNA, and allosteric changes that accompany binding to the ligand. Each of these parameters will have its own fitness function mapped over the same sequence space, creating unique fitness landscapes. An aTF simultaneously traverses these multiple fitness landscapes, which collectively govern the evolutionary trajectory of the aTF under selective pressure. Thus, any one fitness landscape is not adequate as a global measure of transcription factor function. We need to examine multiple fitness landscapes and characterize epistasis in each to understand the evolutionary trajectory of an aTF.

Here, we integrate functional, structural, and biophysical analysis to characterize epistasis in the functional parameters of an allosteric transcription factor. Using computation-guided design, we changed the ligand specificity of TtgR, a microbial aTF, to respond better to one of its native ligands (resveratrol), but not to another (naringenin) by targeting mutations to positions that directly interact with the ligand to create a resveratrol-specific TtgR variant^28,29^. By reconstructing all sequence pathways connecting the two states, we found that nonspecific epistatic interactions of two distinct sets of amino acids separately drive loss of naringenin response while increasing resveratrol response (reporter expression when induced by a ligand). We characterized the fitness landscapes of TtgR in terms of four functional parameters: fold change in gene expression, basal gene expression, maximum gene expression, and sensitivity to the ligand (EC_50_) and showed that although ligand-induced allostery is a composite effect of all four parameters, each parameter shows unique patterns of epistasis, but also notable similarities. The crystal structure of the computationally designed mutant shows that one of the mutations reshapes the binding pocket to favor resveratrol over naringenin through a striking change in its binding orientation while maintaining allostery. We found that epistasis creates distinct biophysical and biological functional landscapes. Our results highlight the functional and evolutionary complexity of allosteric proteins because pathways can traverse through multiple adaptive landscapes under evolutionary pressure^29^. Our approach also provides a general conceptual and methodological framework to investigate epistasis in transcription factors.

## Results

### Computational design of ligand specificity switch

We chose TtgR, a ligand-inducible aTF belonging to the diverse TetR-like protein family, as a target for computational engineering of ligand specificity^29^. TtgR is a 1-component transcriptional system and represents the simplest molecular mechanism for converting biophysical interaction between inducer and protein into a complex biological response like transcription^29^. In the uninduced state, TtgR physically obstructs the RNA polymerase by binding to DNA^29^. When induced, ligand-binding allosterically lowers affinity for DNA, thereby allowing transcription^29,30^. Since TtgR is found in a plant-associated microbe (*Pseudomonas putida*), it is induced by multiple plant molecules including resveratrol and naringenin^28^. Thus, TtgR provides a suitable functional backdrop to investigate the role of epistasis in the emergence of novel function (ligand specificity) in an allosteric protein^28,31^. To emulate the emergence of novel function, we engineered TtgR to respond better to resveratrol and not to naringenin.

We used computational design (Rosetta software suite) to engineer TtgR specificity by generating function-switching mutations that directly interact with the ligand^32^. Less directed approaches may yield a specificity switch, but these can also include distal mutations whose effects on ligand affinity will confound our examination of epistasis^10,33^. Since our goal was to study how local interactions shape global function, computational design was the appropriate tool as in silico mutations are chosen based on interaction energies between protein and ligand^34,35^.

**Fig. 1:**
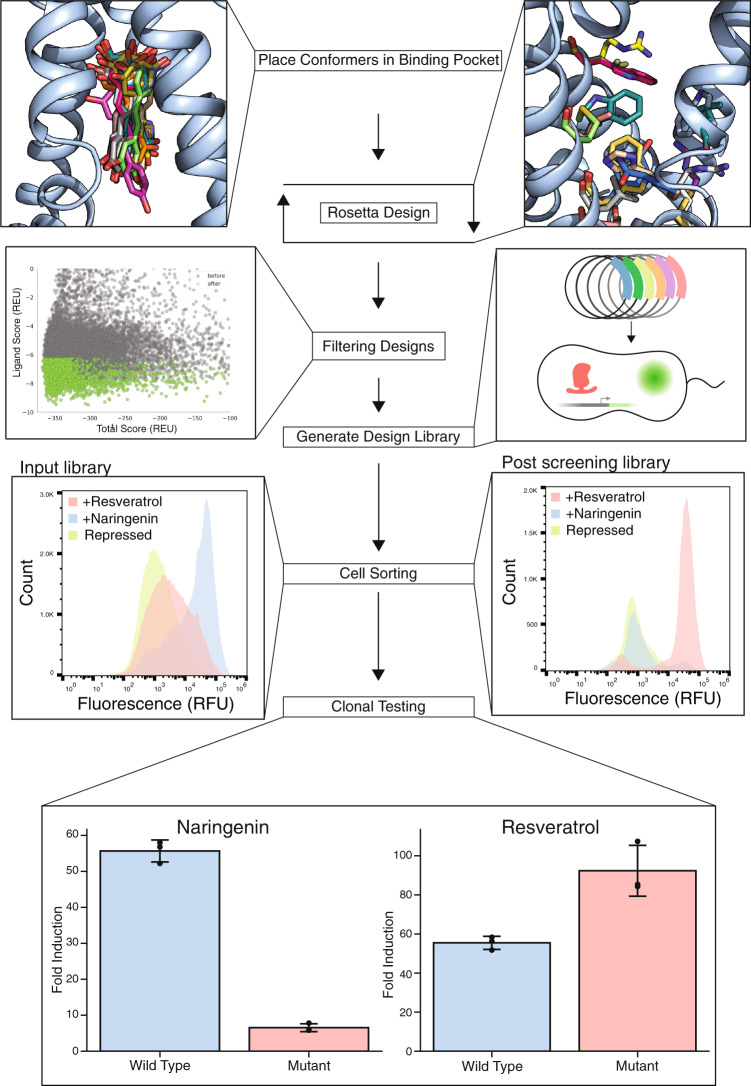
Design of resveratrol-specific TtgR variant. Resveratrol conformers are docked into TtgR followed by Rosetta-based computational design of the binding pocket. Candidates with favorable Rosetta score metrics (green points) are synthesized and cloned into an expression vector. Distribution of fluorescence in cells containing uninduced TtgR variant library (light green), induced with naringenin (light blue) and resveratrol (red) before sorting (Pre-Sort) and after three rounds of sorting (Post-Sort) are shown. Colony screening identified a quadruple mutant showing resveratrol specificity: C137I/I141W/M167L/F168Y. The quadruple mutant phenotype was compared to wild-type in biological triplicate (*n*=3) by inducing each with either 1000 μM naringenin or 100 μM resveratrol. The error bars denote the standard deviation of the fold induction for the triplicate measurements (see “methods”).

To increase resveratrol specificity, we redesigned the ligand-contacting residues for greater affinity for resveratrol, assuming greater affinity may result in greater specificity. Since Rosetta is a structure-based design tool, the absence of a resveratrol-bound TtgR crystal structure made the design task challenging because the correct position of the ligand in the binding pocket was not known a priori. Therefore, we generated a set of diverse starting poses (16) by docking resveratrol conformers in different orientations within the binding pocket (Fig. 1). For each starting pose, we redesigned ligand-contacting residues while permitting constrained rigid-body flexibility of the ligand and torsional flexibility of the protein backbone. We computationally generated approximately 19,000 unique TtgR design variants. After design, each output variant comes with a set of Rosetta-calculated scores that reflect physical properties such as stability, repulsion, hydrogen bonds, and protein-ligand affinity. The best variants for library construction can be selected from the distribution of all scores of output designs based on user-defined preferences. The variants were curated using parameter-specific median absolute deviation cutoffs on a select set of Rosetta scoring metrics to yield a final list of approximately 3500 unique sequences with an average of five mutations per variant for experimental testing (Supplementary Figs. 1, 2). The mutations generated in the 3500 sequences are diverse, but designed sequences generally favor the wild-type amino acid at each mutable position (Supplementary Fig. 2). A few positions such as 96, 137, 168, and 175 have mutations that are more abundant than the wild-type amino acid. We synthesized oligonucleotides encoding approximately 3500 designed variants as a pool of exact chip-DNA sequences (Twist Bioscience Inc).

To determine the activity of TtgR variants, we designed a pooled screen by sorting *E. coli* cells containing a GFP reporter system regulated by a TtgR operator adapted for *E. coli*. We quantified the activity of variants based on fold induction: the ratio of GFP expression with and without inducer. Fold induction is a simple measure of the transcriptional activity of an aTF that accounts for factors affected by epistasis including DNA affinity, ligand affinity and allostery^5,9^. The activity of the initial library was greater toward naringenin than resveratrol with a median fold induction of 21-fold and 2.4-fold with naringenin and resveratrol, respectively (Fig. 1). To enrich resveratrol-specific variants in the library, we devised a toggled screening scheme where we first sorted variants competent for binding to DNA (low GFP with no resveratrol) followed by sorting variants that can activate expression of the reporter (high GFP with resveratrol) (Supplementary Fig. 3). After three rounds of toggled screening, we observed a much greater response to resveratrol than naringenin in the enriched population compared to the input population (Fig.1). From the enriched population, we isolated a resveratrol-specific TtgR variant with four mutations: C137I, I141W, M167L, and F168Y which we will henceforth refer to as the ‘quadruple mutant’. All four mutations were in close proximity to the ligand and no mutations were found elsewhere on TtgR. The quadruple mutant gave 92- and 6.5-fold induction with 100 μM resveratrol and 1 mM naringenin, respectively, compared to 55- and 55-fold of wild-type TtgR (Fig. 1, Supplementary Fig. 4). These concentrations were selected based on the differences in maximum solubility in aqueous solution.

The goal of Rosetta design was to narrow the potential designable sequence space to a subspace of sequences most likely to offer high resveratrol function. It is possible that other Rosetta designs were successful in generating ligand specificity but were lost in the screening process that was engineered to identify only the most successful variants. We found that while the quadruple mutant fell within the cutoffs imposed during the curation process, it was not the best in any scoring parameter. We chose the quadruple mutant as the functional endpoint for characterizing epistasis.

### Epistasis shapes the fitness landscape of resveratrol response

Experimental fitness landscapes are a useful framework for characterizing epistasis by revealing fitness pathways through mutational intermediates that connect two functional states. We constructed multiple fitness landscapes derived from dose–response curves to examine epistatic constraints in the transition from wild-type TtgR to the resveratrol-specific quadruple mutant. We made all single, double, and triple mutation combinations of the four mutations that provide resveratrol specificity as individual clones, resulting in a total of 16 variants (including endpoints). Fitness landscapes are commonly illustrated as a series of nodes and edges. Each node is designated by a binary string in which each number corresponds to a mutable position. A zero indicates the wild-type amino acid identity and a one indicates the substituted amino acid. The positions in order from left to right are 137, 141, 167, and 168 (0000 is wild-type TtgR, 1111 is quadruple mutant, and 0100 represents the I141W mutant).

**Fig. 2:**
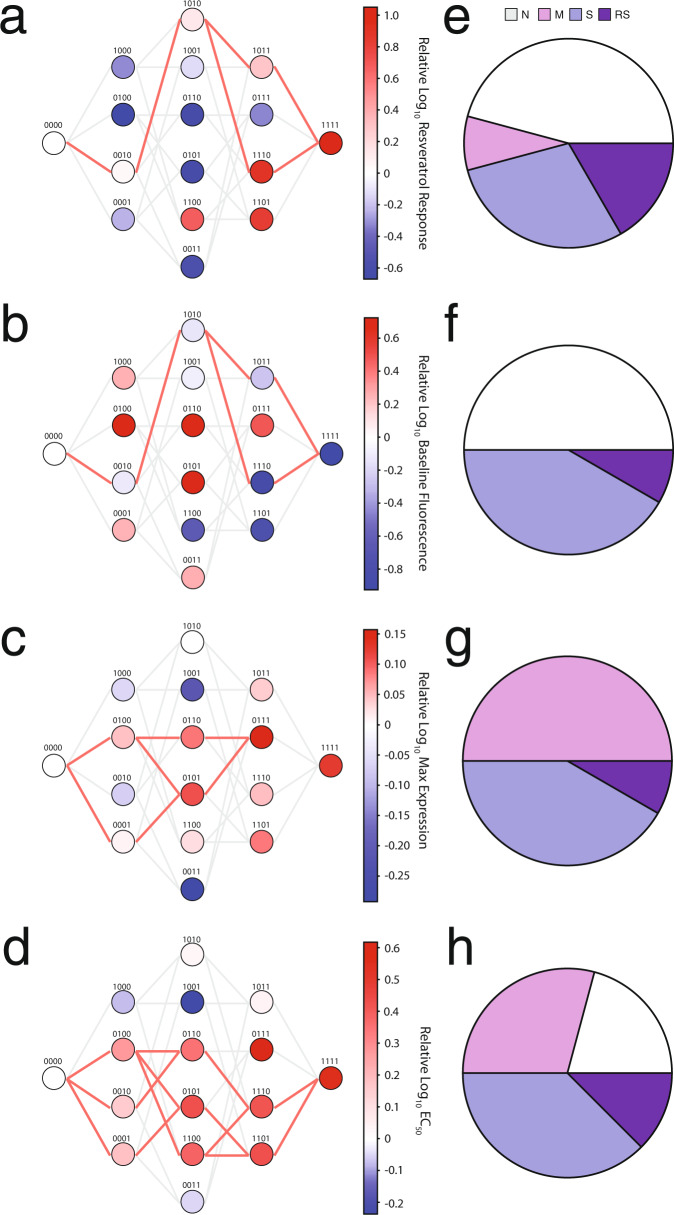
Fitness landscapes for multiple functional parameters in response to induction with resveratrol. Fitness landscapes of (**a**) fold induction, (**b**) basal gene expression, (**c**) maximum gene expression, and (**d**) EC_50_ parameters for all 16 TtgR variants in response to resveratrol with each variant shown as a node in the graph. Each variant is labeled with a binary string corresponding to the presence (1) or absence (0) of a mutation at position 137, 141, 167, or 168 in order. Nodes separated by a single mutation are connected by edges showing viable (bold red) and unviable paths (light gray) through sequence space. Nodes are shaded by log_10_ of the fitness parameter at 250 μM resveratrol normalized to the fitness of wild-type TtgR. Number of epistatic subnetworks in the resveratrol (**e**) fold induction, (**f**) basal gene expression, (**g**) maximum gene expression, and (**h**) EC_50_ landscape determined by Bahadur expansion. Non-epistatic subnetworks (N) are shown in white, magnitude epistasis (M) in pink, sign epistasis (S) in light purple, and reciprocal sign epistasis (RS) in dark purple.

The ability of a transcription factor to control gene expression in response to a small molecule is broadly described by four parameters—(1) fold change in gene expression upon induction (fold induction), (2) basal gene expression without the inducer, (3) maximum gene expression upon induction, and (4) sensitivity to ligand concentration (EC). These parameters capture the mechanistic properties of binding to inducer, binding to DNA, and allosteric communication of ligand binding. To investigate how the same set of binding pocket mutations might uniquely affect each parameter, we constructed the fitness landscape of each parameter individually. We quantified the number of viable pathways in the resveratrol landscape by requiring that each additional mutation must increase parameter fitness if the quadruple mutant performs better than wild type or decrease parameter fitness if the quadruple mutant performs worse than wild type. There are 24 possible pathways from wild type to quadruple mutant (Fig. 2a). Each functional parameter shows distinctive patterns of epistasis, although some are closely related.

In the fold induction landscape, viable pathways must go through 0010 as all other single mutants have lower resveratrol response relative to wild-type TtgR (Fig. 2a). This restricts the number of available pathways from 24 to a maximum of 6. From 0010, there are three possible double mutants: 0011, 0110, and 1010. Both 0110 and 0011 are not viable as their activity substantially decreases compared to 0010 (Fig. 2a). However, 1010 is viable as it gives modestly higher resveratrol response (Fig. 2a). Both C137I and M167L manifest as key permissive intermediates in the fitness landscape that allows I141W (1110) or F168Y (1011) to be added. Since 1010 is the only viable double mutant, the number of available pathways reduces to two (Fig. 2a, bold red lines). Both triple mutants (1011 and 1110) have higher resveratrol response than 1010 which allows two viable pathways to reach the quadruple mutant, which is the global maxima of this fitness landscape (Fig. 2a).

The fitness landscape of basal gene expression resembles the fold induction landscape, with identical viable pathways, as the nodes with lower basal gene expression also show higher fold induction. All the nodes along viable pathways have lower basal gene expression than wild-type TtgR (0000) and the quadruple mutant is one of the mutants with lowest basal gene expression (Fig. 2b). The adaptive landscapes of maximum gene expression and EC_50_ show similar features to each other including a general trend of increasing magnitude from 0000 to 1111 (Fig. 2c,d). Since the global maxima for maximum gene expression is 0111 (not 1111), all pathways on the maximum gene expression landscape terminate at 0111 (Fig. 2c). Six pathways are allowed in the EC_50_ landscape because of the general tendency of mutations to increase EC_50_ regardless of mutational background (Fig. 2d). There is an interesting dependence between maximum gene expression and EC_50_ where nodes with high expression tended to also have high EC_50_ (low ligand sensitivity), indicating a likely trade off where high gene expression comes at the expense of ligand sensitivity. In other words, it may be difficult to achieve an ultrasensitive response concomitantly with a large change in gene expression. Since small deviations in activity may be permitted during evolution, we relaxed the requirement that each subsequent step through sequence space change fitness to be more like the quadruple mutant. We allowed small losses in the function of 25% between nodes and found that additional pathways are tolerated in the basal gene expression, maximum gene expression, and EC_50_ landscapes. No additional pathways exist in the resveratrol fold induction landscape (Supplementary Fig. 5).

Next, we delved deeper into the key epistatic interactions that shape the fitness landscapes. Epistatic interactions are classified as magnitude, sign, or reciprocal sign based on the combined effect of a pair of mutations relative to the effect of each mutant individually. Magnitude epistasis occurs when both mutations individually are beneficial or detrimental and their combined effect is greater in magnitude than the sum of their individual effects (Supplementary Fig. 6). Sign epistasis occurs when the effect of one mutation switches from beneficial to deleterious or vice versa depending on if the other mutation is present (Supplementary Fig. 6). Reciprocal sign epistasis occurs when both mutations switch effects when paired (Supplementary Fig. 6).

Two epistatic interactions, C137I-I141W and M167L-F168Y, play important roles in modulating basal gene expression and fold induction. C137I mutation makes epistatic interactions with all the other three mutations (1100, 1010, or 1001) which are critical to control basal gene expression through sign or reciprocal sign epistasis (Fig. 2b). This is best exemplified by the interaction between C137I (1000) and I141W (0100) in the basal gene expression landscape. Both 1000 and 0100 have high basal gene expression while the double mutant 1100 has low basal gene expression leading to reciprocal sign epistasis. This interaction shows mutations in the binding pocket trigger the allosteric network to create new nonspecific epistatic interactions at a distal site (in this case, the DNA-binding interface). The other double mutants that contain C137I (1010 and 1001) also have decreased basal gene expression, which is maintained through the quadruple mutant by non-epistatic (1100-1111, 1010-1111, and 1001-1111) interactions (Fig. 2b). The I141W mutation is also a key modulator of fold induction that manifests through controlling basal gene expression. Although this mutation by itself causes high basal gene expression (low fold induction) when paired with either M167L (0110) or F168Y (0101) in any combination, in the 1100 background both M167L (1110) and F168Y (1101) have low basal gene expression (high fold induction) and form a magnitude epistasis interaction to generate the phenotype of the quadruple mutant (Fig. 2b).

The M167L mutation makes a strong nonspecific epistatic pair with the F168Y mutation, creating a reciprocal sign epistasis interaction in the EC_50_ landscape and sign epistasis in the basal gene expression, maximum gene expression, and fold induction landscapes. In the EC_50_ landscape, M167L is the only node that decreases EC_50_ that does not contain C137I (Fig. 2d). However, this effect is masked by the addition of either C137I or I141W. The two mutations show sign epistasis in the maximum gene expression landscape in the C137I background (1000-1011) and magnitude epistasis in the I141W or C137I-I141W background, indicating that the pair behavior is dependent on the background mutations (Fig. 2c).

While a qualitative description of epistasis is easy to visualize, we wanted to also quantify the extent of and characterize the type of epistasis within all individual subnetworks and the entire 16-variant system.  A subnetwork is a set of four variants comprising a background variant, two single mutants and a double mutant introduced into the background variant. We used Bahadur expansion to describe all pairwise and higher order interactions (see “Methods”)^36^. The Bahadur expansion models the activity of the landscape using a linear sum of interaction terms and coefficients. Orders of interactions (first [solo], second [pairwise], third [three way], or fourth [four way]) can be included in this sum to understand their contribution to modeling the behavior of all variants. For each subnetwork, we computed the correlation coefficient between a linear sum of first-order interaction terms and actual experimental data. In the simplest case of no epistasis, the correlation coefficient of this comparison (R^2^) is close to 1, but any deviation (*R*^2^ < 1.0) indicates the prevalence of epistasis. We applied the Bahadur expansion to quantify epistasis in all 24 subnetworks for each fitness parameter. The fitness landscapes of basal gene expression, maximum gene expression, and EC_50_ had unique patterns of epistatic interactions (Fig. 2e–h).

Epistasis thus has a large role in shaping the fold induction landscape between the wild type and the quadruple mutant through key interactions. The magnitude and location of the epistatic interactions are unique to their respective fitness property. Although the global expansion first-order terms explain the majority of the variance in the fold induction landscape, higher order epistatic interactions influence resveratrol fold induction by modulating interactions in secondary and tertiary subnetworks to improve the resveratrol response (Supplementary Fig. 7).

### Epistasis uniquely influences the fitness landscape of each ligand

As inducer specificity changes, the fitness landscape of the same mutational intermediates will differ for each inducer. These differences may reveal alternative adaptive pathways in the fitness landscape of one inducer that circumvent functional “dead ends” in the fitness landscape of another inducer. Therefore, we examined the fitness landscape of naringenin-induced response by evaluating the same four parameters: fold induction, basal gene expression, maximum gene expression (at 2000 μM), and EC_50_ of all 16 variants for comparison with the fitness landscapes of resveratrol. We determined the number of viable pathways by requiring that each additional mutation must have a change in fitness that bridges wild type and the quadruple mutant to emulate the progressive change in function during evolution.

**Fig. 3:**
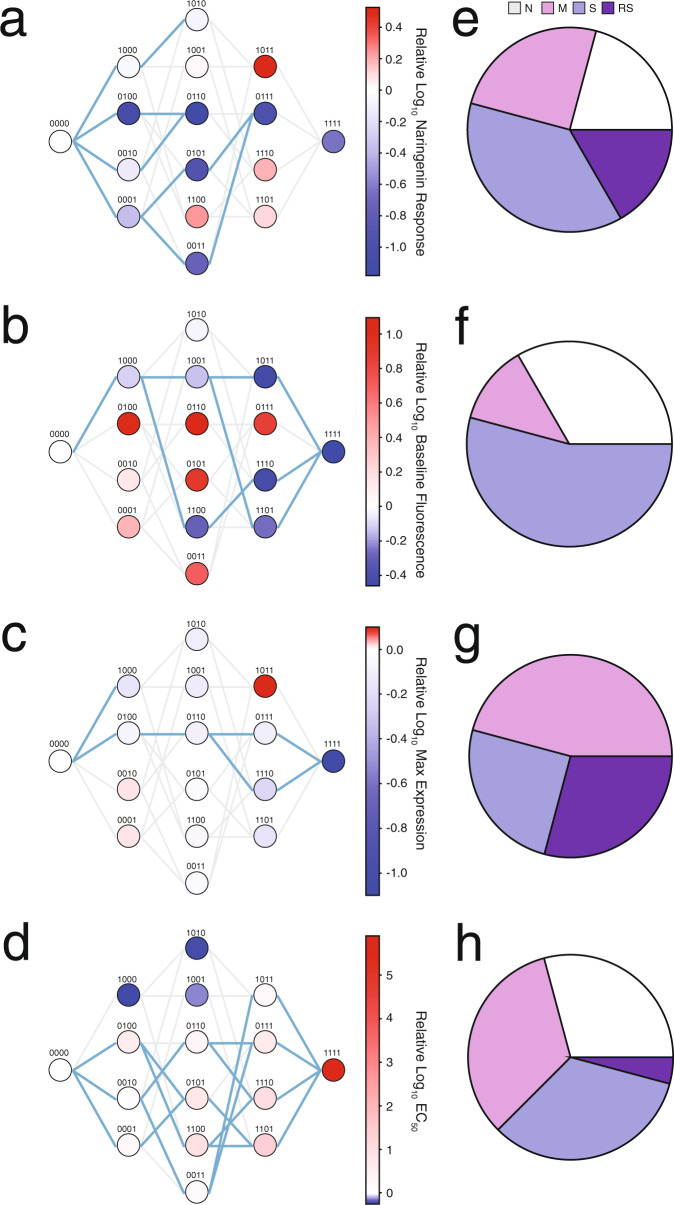
Fitness landscapes for multiple functional parameters in response to induction with naringenin. Fitness landscapes of (**a**) fold induction, (**b**) basal gene expression, (**c**) maximum gene expression, and (**d**) EC_50_ parameters for all 16 TtgR variants in response to naringenin with each variant shown as a node in the graph. The labeling and line colors are analogous to Fig. 2. Nodes are shaded by log_10_ of the fitness parameter at 2000 μM naringenin normalized to the fitness of wild type TtgR. Number of epistatic subnetworks in the resveratrol (**e**) fold induction, (**f**) basal gene expression, (**g**) maximum gene expression, and (**h**) EC_50_ landscape determined by Bahadur expansion. The labels of the pie charts are identical to Fig. 2.

In the fold induction landscape, none of the 24 possible pathways viably connect wild type to quadruple mutant because the global minima (variant with lowest naringenin response) in the landscape is the double mutant 0110, not the quadruple mutant (1111) (Fig. 3a). In the basal gene expression landscape, three pathways connect wild type to the quadruple mutant through the C137I (1000) mutation (Fig. 3b). Pathways emerging from 1000 pass through two double mutants, 1001 and 1100, with lower basal gene expression. The basal gene expression of 1001 is higher than 1100, allowing 1001 to link to both triple mutants (1011 and 1101) compared to the single triple mutant from 1100 (1110). The maximum gene expression landscape contains two pathways connecting wild type to quadruple mutant (Fig. 3c). Although many nodes have lower maximum gene expression compared to the preceding node, most are not part of pathways that bridge wild type and the quadruple mutant. Two single mutants (1000 and 0100) have lower maximum gene expression than wild type, but only one is connected to a viable double mutant (0110). Both triple mutants (0111 and 1110) accessible from 0110 connect to the quadruple mutant. Like the EC_50_ landscape of resveratrol, the EC_50_ landscape of naringenin is characterized by a general increase in EC_50_ as mutations accumulate (Fig. 3d). There are eight possible pathways that link wild type to the quadruple mutant. Three of the four single mutants increase EC_50_ (0100, 0010, and 0001). Four of the double mutants and all the triple mutants are accessible by at least one of the preceding nodes, but not every double or triple mutant is accessible from all preceding nodes due to minor deviations in the general trend of increasing EC_50_. No additional mutational pathways are tolerated even when increases of up to 25% naringenin response are allowed between nodes for the naringenin fold induction landscape (Supplementary Fig. 8). Similarly to the resveratrol landscapes, the basal gene expression, maximum gene expression, and EC_50_ landscapes show additional pathways at this tolerance.

Closer examination of the role of individual mutations shows that C137I and I141W have strong effects on multiple landscapes. C137I (1000) is the only mutation that decreases EC_50_ relative to wild type (Fig. 3d). Two additional double mutants 1010 and 1001 further decrease EC_50_ but pairing C137I with I141W (1100) or C137I with both M167L and F168Y (1011) increases EC_50_, suggesting that these mutational combinations may mask the effect of C137I. As with the resveratrol landscapes, the I141W mutation has an important role in modulating basal gene expression and fold induction (Fig. 3a, b). Any mutant containing I141W, but not C137I has higher basal gene expression (lower folder induction) than wild type. Combining I141W and C137I results in a large decrease in basal gene expression, which further decreases upon the addition of either M167L (1110) or F168Y (1101). M167L and F168Y individually result in incremental changes in basal gene expression, maximum gene expression, and EC_50_ (Fig. 3b–d). However, the M167L-F168Y double mutant shows interesting context-dependent effects due to nonspecific epistasis. For example, in the fold induction landscape, the combination of M167L and F168Y is beneficial in 1000 background but is detrimental in the 1100 background (Fig. 3a). This dependent behavior extends to all the other fitness landscapes even though the mutational background and types of epistasis change.

Epistasis shapes the fitness landscape of each function (naringenin and resveratrol response) in distinct ways. Furthermore, each functional parameter (basal gene expression, maximum gene expression, or EC_50_) is affected uniquely by the addition of multiple combinations of mutations. I141W controls high basal gene expression and strongly modulates fold induction regardless of ligand. In contrast, C137I is more context-dependent; it is responsible for low EC_50_ values solo or in combination with either M167L or F168Y in the naringenin landscape but is strongly influenced by M167L in the resveratrol EC_50_ landscape. Some epistatic pairs are consistent between the resveratrol and naringenin landscapes. The C137I + I141W pair strongly affects basal gene expression and fold induction for both ligands. The M167L + F168Y pair has unique behavior in all fitness landscapes that is dependent on the mutation background into which they are introduced. However, the pair’s effect on the wild type background is stronger in resveratrol compared to naringenin for all parameters. The patterns of epistasis in the naringenin subnetworks are unique to their respective functional parameter (Fig. 3e–h). Furthermore, the same set of mutations that create epistatic interactions giving rise to high resveratrol response forge ligand-specific epistatic patterns in the fold induction, basal gene expression, maximum gene expression, and EC_50_ landscapes (Supplementary Fig. 9).

### Crystal structure reveals molecular basis of specificity of quadruple mutant

**Fig. 4:**
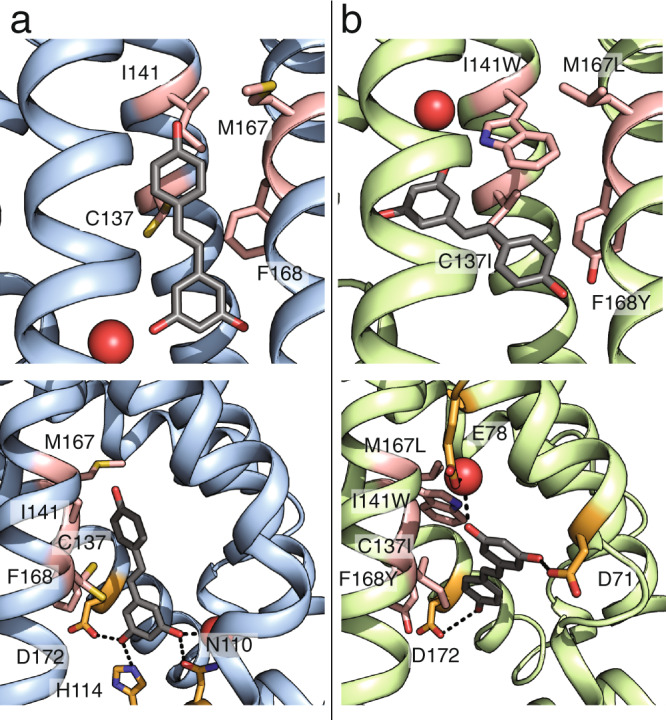
Structural basis for ligand specificity. Wild-type TtgR and quadruple mutant are shown in blue and green ribbons, respectively. Positions 137, 141, 167, and 168 are colored in pink. Resveratrol is shown as gray sticks. Water molecules are shown as red spheres. **a** Binding pocket of resveratrol-bound wild-type TtgR (PDB ID: 7K1C) (upper panel) with residues making hydrogen bonds to resveratrol highlighted in orange (lower panel). **b** Binding pocket of resveratrol-bound quadruple mutant TtgR (PDB ID: 7KD8) (upper panel) with residues making hydrogen bonds to resveratrol highlighted in orange (lower panel).

To understand the structural basis of TtgR-ligand interactions, we solved high-resolution crystal structures of quadruple mutant (resveratrol-bound and apo) and wild-type TtgR (resveratrol-bound) at a resolution of 1.9 Å or better (Supplementary Table 3). TtgR is a compact, dimeric, all-helical transcription factor with a large cavity between five angled helices forming the ligand-binding pocket (Supplementary Fig. 10a, b). The quadruple mutant bound to resveratrol (PDB: 7KD8) is structurally very similar to the wild type with an all-atom RMSD of 1.2 Å over the entire structure. The DNA-binding domains of the resveratrol-bound quadruple mutant and the resveratrol-bound wild type are extremely similar with an all-atom RMSD of 1.0 Å (Supplementary Fig. 11). The four mutations do not substantially change the volume of the pocket (215Å^3^ in wild type compared to 234 Å^3^ in the quadruple mutant) or the surface area of the pocket (184 Å in wild type compared to 186 Å in the quadruple mutant) (Supplementary Fig. 12). The position and orientation of resveratrol in the wild-type TtgR structure (PDB: 7K1C) resembles the position and orientation of naringenin in a previously solved co-crystal structure of TtgR (PDB: 2UXU)^28^. In both structures, the ligands bind in a vertical mode such that the plane of the molecule is roughly perpendicular to DNA (Supplementary Fig. 10c). In wild-type TtgR, the four mutated positions (C137, I141, M167, and F168) are located approximately in the center of the binding pocket and make nonspecific van der Waals interactions with resveratrol (Fig. 4a, upper panel). Other neighboring residues N110, D172 and H114 make specific hydrogen bonds that stabilize resveratrol in the vertical orientation (Fig. 4a, lower panel). Although both naringenin and resveratrol bind in the vertical orientation, only N110 is able to make a hydrogen bond with both naringenin and resveratrol^28^. The ability of wild-type TtgR to bind multiple ligands likely arises from the nonspecific interactions made by the nonpolar amino acids in the binding pocket.

The structure of the quadruple mutant reveals the role of individual residues in ligand specificity. I141W, a mutation critical for resveratrol specificity, creates a large steric barrier that alters the shape of the pocket and obstructs the vertical binding orientation of ligands (Fig. 4b, upper panel). Resveratrol is accommodated in the binding pocket in a horizontal binding orientation almost parallel to the plane of the tryptophan. Unlike I141W which plays a clear steric role, the other three mutations (C137I, M167L and F168Y) have a more subtle effect in reshaping the binding pocket through nonpolar interactions. C137I mutation creates a protrusion in the binding pocket that increases shape complementarity to resveratrol (Supplementary Fig. 13a). M167L is buried between the residues in the binding pocket and the dimerization helix and may play a role in positioning the I141W tryptophan to stabilize its horizontal orientation through van der Waals interactions (Supplementary Fig. 13b). F168Y allows the formation of multiple hydrogen bonds with nearby water molecules and may serve to stabilize the structure (Supplementary Fig. 13b). A different hydrogen bonding network consisting of D71, R75, and E78 make hydrogen bonds with the resveratrol molecules in chain A (Supplementary Fig. 13c) and D71, E78, D172, and a nearby water molecule make a hydrogen bond with the single resveratrol molecule in chain B (Fig. 4b, lower panel).

Although resveratrol and naringenin share similar chemical backbones, naringenin is bulkier than resveratrol due to the fused carbon rings of the chromanone. This reduces the shape complementarity of naringenin to the redesigned binding pocket despite the similarity in the volume of the quadruple mutant and wild-type binding pockets (Supplementary Figs. 12, 14). The 4-hydroxyphenyl moiety and the carbonyl group of the 4-chromanone backbone of naringenin could create steric clashes with residues lining the wall of the pocket and cause the ligand to sample less space in the pocket compared to resveratrol, which provides a reasonable structural basis for ligand specificity.

The new binding mode of the quadruple mutant was not predicted in the original design scheme. We seeded the input structures for the Rosetta design with resveratrol docked in the vertical orientation to mimic the binding mode of the wild-type structure. The design process is only able to make minor alterations to the position and angle of the ligand in the binding pocket (Supplementary Fig. 15). However, Rosetta was able to identify a subset of positions that, when mutated, could confer resveratrol specificity.

The structural basis of ligand specificity relies on the I141W substitution to create a steric barrier to prevent binding in the vertical orientation, which is observed in wild-type TtgR for multiple ligands. In the novel horizontal mode, other ligands may be occluded from the pocket through steric clashes with wild-type residues in the pocket. The epistatic interactions observed in the fitness landscapes for naringenin and resveratrol can be rationalized through examination of the structure. The C137I-I141W pair increases shape complementarity to resveratrol while M167L-F186Y contact the dimerization helix and potentially affect the positioning of nearby residues that interact with the ligand. The altered binding mode establishes that allostery is robust to major changes in the binding mode in TtgR.

### Relationship between biophysical affinity and biological response

Ligand response of an aTF is a complex combination of both biophysical interactions and allostery. Mutations that affect aTF fold induction can do so by altering ligand affinity, DNA affinity, or the allosteric signal upon ligand binding. Since all four mutations are localized to the binding pocket, the observed changes in fold induction of TtgR are likely due to altered binding affinity to ligand, the transmission of allosteric signal, or both. To understand the relationship between biophysical affinity and biological response, we compared changes in ligand affinity (K_d_) to changes in ligand sensitivity (EC_50_) for both naringenin and resveratrol. We chose mutants in the 0000-1000-0100-1100 subnetwork because it is important for the high resveratrol response in the quadruple mutant. Further, this network shows a strong manifestation of nonspecific epistasis through reciprocal sign change and is therefore a good model to understand the relationship between biophysical affinity and biological response. We estimated ligand affinity using isothermal titration calorimetry (ITC) of purified proteins and ligand sensitivity from dose–response curves. Ligand sensitivity is derived from reporter expression and is thus a combination of both allostery and affinity.

**Fig. 5:**
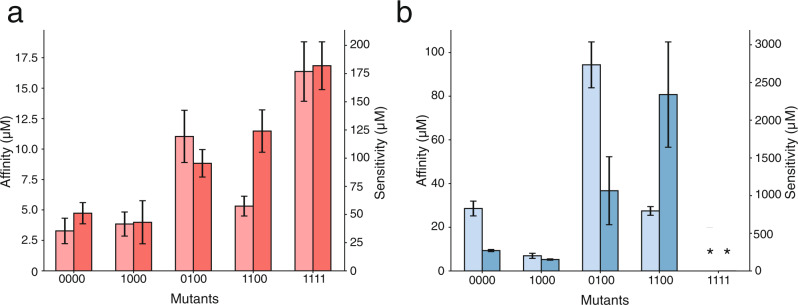
Comparison of biophysical and biological properties of TtgR variants. Ligand affinity (light bar) and EC_50_ sensitivity (dark bar) for resveratrol (**a**) and naringenin (**b**) are shown for TtgR variants 0000, 1000, 0100, 1100, and 1111. Ligand affinity was estimated by isothermal calorimetry and EC_50_ sensitivity from fitting dose–response curves to the Hill equation. EC_50_ values and error are calculated based on fitting to triplicate dose–response curves. ITC values and error are generated from a one-site binding model; error bars denote the error of the fit (see methods). The values shown are likely to underestimate the actual affinity and sensitivity values.

Affinity and sensitivity of resveratrol for different variants are generally concordant for resveratrol, with the exception of 1100 (Fig. 5a). We note that the ITC and dose–response curves for some variants did not plateau due to poor ligand solubility at high concentrations resulting in imprecise estimates of K_d_ and EC_50_. Nonetheless, qualitative comparisons can be made to gain useful insight. For instance, comparison of ITC profiles of 0000 and 1111 for resveratrol shows weaker binding for 1111 even though the precise K_d_ may be difficult to measure. Similarly, dose–response curves show weaker EC_50_ for 1111 than 0000 even though it is not fully saturated. The C137I mutation appears to be largely responsible for the affinity in 1100, but the I141W mutation causes the increase in sensitivity. In general, as mutations accumulate from wild type, the affinity and sensitivity generally decrease, suggesting a decreased ability to undergo allosteric changes is likely due to weaker binding (Fig. 5a). The discordance between affinity and sensitivity is much greater for naringenin than resveratrol. In the case of naringenin, no relationship was evident between affinity and sensitivity across the subnetwork (Fig. 5b). Although the quadruple mutant has higher resveratrol fold induction than wild type, its affinity and sensitivity for resveratrol are lower than that of wild type (Figs. 1, 5a). In essence, these examples illustrate the complex relationship between local interactions (specific epistasis) and their global effects in allosteric proteins.

The 0000-1000-0100-1100 subnetwork displays a unique, ligand-specific pattern of specific epistasis for biophysical and biological parameters. The mutations we introduced into TtgR suggest an effect on allostery changes in EC_50_ as the complexities of function may not be simply explained by changes in biophysical affinity. These measurements also suggest that by optimizing a particular protein function (fold induction), other parameters (sensitivity or affinity) may not necessarily stay at fitness maxima as the 1111 mutant shows poor sensitivity (high EC_50_) to both ligands.

## Discussion

In this study, we describe the pervasive effects of epistasis on ligand specificity in a simple allosteric transcription factor by examining fold induction, basal gene expression, maximum gene expression, and EC_50_ of two ligands across multiple mutants. By leveraging computational protein design, we engineered four mutations into TtgR, a transcription factor that can normally bind to both resveratrol and naringenin, to only bind to resveratrol. By characterizing the functional response to both resveratrol and naringenin across all combinations of mutations, we show that the extent of epistasis between mutations affecting multiple protein functions is specific for each ligand. For instance, 50% of subnetworks meet the criteria for epistasis for resveratrol fold induction while 83% of subnetworks are epistatic for naringenin fold induction. However, the fitness landscapes of both ligands are shaped by common critical pairs of epistatic interactions (C137I and I141W or M167L and F168Y), though their behavior may be different depending on the functional parameter. The biological effects of these mutations are further validated by the crystal structures. The four mutations localize to one face of the binding pocket, making nonpolar interactions with the ligand. C137I and I141W increase shape complementarity of the pocket for resveratrol, but only in an alternative horizontal binding pose. The four mutations that confer ligand specificity decrease both affinity and sensitivity suggesting that the changes in sensitivity could be a consequence of lower affinity and not necessarily a purely allosteric effect.

Our study used a constrained set of mutations chosen through in silico selection as opposed to the selection of random mutations through natural evolution. An evolutionary process may have selected a different set of mutations to confer the same functional outcome, leading to the presence of a different pattern of epistasis for either naringenin or resveratrol response. Often in natural evolution, mutations that are distal to the site of interest have a profound effect on protein function^8,21^. These background mutations complicate any examination of key mutations within the targeted area of the protein and their influence on protein function. By utilizing a combination of computational design and high-throughput screening, we targeted mutations to a discrete set of ligand-interacting positions within the binding pocket. Our approach enabled us to examine the propensity of epistasis in a constrained setting where mutations are limited to those that interact directly with the ligand, enabling the examination of the intersection of mutation, biophysical epistasis, and biological epistasis.

Our results highlight the dependence of epistasis on protein function and the prevalence of distinctive adaptive landscapes for multiple functions within the same set of mutations. This process highlights the functional tradeoffs that occur during an evolutionary process and raises the implication that proteins with multiple functions may readily traverse nonoptimal sequence space through varying selective pressures. These landscapes can thus become interconnected by changing selection pressures between different protein functions. On an evolutionary scale, simultaneously changing protein sequence and selection pressure may enable improbable trajectories by bypassing epistatic barriers to reach previously inaccessible mutational states. In our case, higher order epistasis which prevents access to the quadruple mutant in the naringenin fold induction landscape, could be bypassed by toggling between naringenin and resveratrol selection pressures. The evolution of allosteric proteins is inherently dependent on epistasis and the interactions arising between mutations in these proteins uniquely affects multiple adaptive landscapes.

## Methods

### Computational design

Protein modeling and design was performed with Rosetta version 3.5 (2015.19.57819)^35,37^. Python and shell scripts for generating input from Rosetta and analyzing from Rosetta are available at: https://github.com/raman-lab/biosensor_design

The high-resolution TtgR structure co-crystalized with tetracycline was selected as the starting point for computational design (PDB: 2UXH)^28^. The structure was prepared for use in Rosetta by performing an all-atom, coordinate-constrained relaxation^38^.

Rosetta/main/source/bin/idealize_jd2.linuxgccrelease -database Rosetta/main/database/ -in::file::fullatom -s 2UXH.pdb -extra_res_fa LG.params -no_optH false -flip_HNQ

Rosetta/main/source/bin/relax.linuxgccrelease -database Rosetta/main/database/ -relax::sequence_file always_constrained_relax_script -constrain_relax_to_native_coords -relax::coord_cst_width 0.25 -relax::coord_cst_stdev 0.25 -s 2UXH_idealized.pdb -in::file::native 2UXH_idealized.pdb -extra_res_fa LG.params -in::file::fullatom -no_optH false -flip_HNQ

Rosetta/main/source/scripts/python/public/molfile_to_params.py -n resveratrol.params -p resveratrol.pdb

The RosettaScripts protocol used to design the ligand-binding pocket of each starting TtgR-resveratrol complex was based on enzyme design protocols^32,39^.

Rosetta/main/source/bin/rosetta_scripts.linuxgccrelease -database Rosetta/main/database/ -parser::protocol enzdes.xml -in::file::s 2UXH_resvertrol.pdb -extra_res_fa resv.params -use_input_sc -packing:linmem_ig 10 -ex1-ex2 -run:preserve_header -enzdes_out -enzdes:bb_min_allowed_dev 0.2 -enzdes:loop_bb_min_allowed_dev 0.5 -enzdes:minimize_ligand_torsions 15 -parser::script_vars ligchain = X resfile = TtgR.resfile -out::pdb -nstruct 10

The TtgR.resfile is a plain text file containing the amino acid position numbers that were able to be mutated during design, and these were positions 137, 141, 167, 168, 171, 172, 175, and 176. We used UW-Madison’s Center for High Throughput Computing computer cluster to perform 320,000 different design simulations. The resulting designed structures were curated to yield the set of sequences that we synthesized to isolate resveratrol-specific TtgR variants.

We selected computational designs for synthesis by first removing designs that were repetitive and then removing designs that were energetically unfavorable. The criteria for unfavorable energies were selected empirically based on the distribution of energies for all designs to yield approximately 10^4^ sequences for synthesis. Specifically, on each unique design, ∆∆G stability calculations were performed on designed residues to ensure the number of destabilizing changes was limited. If the mutation destabilized the TtgR-resveratrol complex by 0.5 Rosetta Energy Units (REU), the residue was reverted to its wild-type identity. After this, non-unique designs were again removed. The unique designs were filtered using distance from the median absolute deviation of several salient Rosetta scoring metrics including total ligand binding energy, hydrogen bond energy, Leonard-Jones repulsive energy, solvation energy, and total score, which is a weighted, linear combination of all score terms in the energy function^34^. Designs that passed this filter were synthesized for library screening../biosensor_design/fas_from_pdb_stdout.py *.pdb > TtgR_resveratrol_all_designs.fasta./biosensor_design/uniquify_fas.py TtgR_resveratrol_all_designs.fasta > TtgR_resveratrol_unique_designs.fasta./ddg_monomer.static.linuxgccrelease -database./database @ddg_flags -in:file:s design_pdb.pdb -ddg::mut_file list_of_positions_to_calc_ddg.mutfile -ddg::iterations 50./gen_enzdes_cutoffs.py concatentated_design_score_file.sc -c median_abolute_deviation_cutoffs.txt -o designs_passing_filter.sc

The median absolute deviation cutoffs used were:

total_score < +1 MAD

fa_rep < +3 MAD

hbond_sc < +3 MAD

tot_burunsat_pm < +3 MAD

%(LIG)s_fa_rep < +3 SD

%(LIG)s_hbond_sc < +3 MAD

%(LIG)s_burunsat_pm < 2.5 ABS

%(LIG)s_total_score < −1 MAD

### Library synthesis

The sfGFP reporter plasmid was constructed using a backbone containing the ColE1 origin and a kanamycin resistance gene. The TtgR operator sequence was modified to contain canonical −10 (5′-TATAAT-3′) and −35 (5′-TTGACA-3′) elements in the promoter. A strong RBS (g10) was chosen for high sfGFP expression^40^. The TtgR operator-RBS sequence was constructed via sequential PCR reactions with overlapping primers containing homology to the pColE1 backbone 5′ of sfGFP (Supplementary Table 1). The plasmid was annealed using isothermal assembly using 0.16pmol of backbone and 0.43pmol of promoter^41^. DH10B cells (NEB) were transformed with the pColE1 reporter plasmid and plated on LB-kanamycin agar (50 μg/mL). A colony was selected and grown in LB-kanamycin media (50 μg/mL) shaking for 16 h at 37 °C. An aliquot of the culture was stored at −80 °C in 25% glycerol. Plasmids were isolated using a DNA miniprep kit (Omega BioTek) according to the manufacturer’s protocol. The insertion of TtgR operator sequence was confirmed via Sanger sequencing.

The TtgR expression plasmid used the SC101 origin and a spectinomycin resistance gene. The constitutive promoter-RBS combination apFAB61-BBa_J61132 and the *TtgR* gene were amplified via KAPA HiFi PCR mix (Roche) using primers with homology to the pSC101 backbone^42^. The *TtgR*-pSC101 construct was generated using isothermal assembly (0.046pmol backbone and 0.24pmol *TtgR*) and DH10B cells were transformed with the *TtgR*-pSC101 construct. A colony was selected and grown in LB-spectinomycin media (50 μg/mL) shaking for 16 h at 37 °C. An aliquot was stored at −80 °C and plasmids were isolated and verified as described previously.

Rosetta-designed sequences were synthesized as exact oligos (Twist Biosciences). Oligos were converted to double-strand DNA using qPCR and purified on a spin column (EZNA Cycle Pure kit from Omega BioTek). The pSC101 backbone was amplified with two separate primer pairs encoding BsaI cut sites that matched the insertion location of the oligos on the *TtgR* gene. The amplified backbone was treated with Dpn1 for 16 h at 37 °C (NEB) followed by a purification using a spin column. The backbone was treated with BsaI (NEB) for 2.5 h at 37 °C followed by purification using a spin column. The digested backbone was treated with Antarctic phosphatase (NEB) for 1 h at 37 °C followed by purification using a spin column. A golden gate reaction (NEB) was performed using 0.12pmol backbone and 0.89pmol library oligo in roughly a 1:7 molar ratio and incubating for 30 cycles of 37 °C for 5 min and 16 °C for 5 min followed by 60 °C for 5 min. A control reaction was made using just the pSC101 backbone with no Rosetta oligos added. The golden gate reactions were dialyzed using semi-permeable membranes (Millipore) for 1 h at 25 °C against dH_2_O. 25 μL of C3020 cells (NEB) were transformed with 2 μL of the dialyzed golden gate mixture via electroporation. Cells recovered for 1 h in SOC media shaking at 37 °C and were diluted 5X with LB. Dilutions of 100X, 500X, and 1000X were plated to calculate transformation efficiency relative to the control. A transformation was considered successful when CFU/mL of the Golden Gate reactions exceeded CFU/mL of control reactions by a factor of 10 or more. Cells grew for 6 h post-transformation before the culture was diluted 50X and grown overnight shaking at 37 °C for 16 h. Plasmids of the library were harvested using a DNA miniprep kit and stored at −20 °C.

An aliquot of the pColE1 frozen stock was streaked on a LB-kanamycin agar plate and grown for 16 h at 37 °C. A single colony was selected and grown in LB-kanamycin media shaking for 16 h at 37 °C. The culture was diluted 50X and grown at 37 °C to an OD_600_ of 0.6. Cells were placed on ice and 5 mL aliquots were centrifuged at 5,500 *g* for 5 min at 4 °C. Pellets were resuspended, washed with ice cold dH2O, and spun at 5,500 *g* twice. The cells were resuspended in 20 μL of water to create electrocompetent DH10B containing the pColE1 plasmid. DH10B *E.coli* containing the pColE1 reporter plasmid were transformed with the initial Rosetta library in pSC101 via electroporation. The transformed cells were recovered for 1 h shaking at 37 °C before dilutions were plated on LB-kanamycin/spectinomycin agar plates (50 μg/mL each) to calculate transformation efficiency. The remaining cells were diluted 5X with LB- kanamycin/spectinomycin media and grown shaking at 37 °C for 16 h. A frozen stock was made with 25% glycerol.

Fifty microliters of aliquots of the cotransformed Rosetta libraries were thawed on ice and inoculated into 5 mL of LB-kanamycin/spectinomycin and grown shaking at 37 °C to an OD_600_ of 0.2. Wild-type cotransformed *TtgR* sensor + reporter was also inoculated as a reference. These were then split into 4 1 mL aliquots and inoculated with either 500 μM naringenin (DMSO), 95 μM resveratrol (ethanol), DMSO, ethanol and grown for 14 h at 37 °C shaking. Cells were diluted 50X in ice cold PBS (137 mM NaCl, 2.7 mM KCl, 10 mM Na_2_HPO_4_, 1.8 mM KH_2_PO_4_) and stored on ice prior to sorting.

Sorting was conducted using a Sony SH800 cell sorter. Cells were excited by a 488 nm laser and GFP fluorescence was captured through a 525/50 filter. Gain settings were adjusted such that all cells fell between 10^2^ and 10^6^ RFU. 100,000 event measurements of all libraries, induced and repressed, were taken to draw gates according to population percentage.

Sorting followed an induced-repressed schema; the first library sort consists of taking 500,000 cells of median 50% of fluorescence from the nontreated distribution (Supplementary Fig. 16). This sort isolates cells that contain TtgR variants capable of repressing *GFP* expression. Cells were sorted into 2 mL of LB. LB as added to a final volume of 5 mL and incubated for 1 h at 37 °C shaking. Kanamycin and spectinomycin were added after 1 h to a final concentration of 50 μg/mL each from 1 mg/mL stocks. These grew to an OD_600_ of 0.2 before frozen stocks were made in 25% glycerol. A small aliquot was stored as a frozen stock at −80 °C in 25% glycerol. The remaining culture was induced with naringenin, resveratrol, DMSO, or ethanol at an OD_600_ of 0.2.

The next sort consisted of isolating 100,000 cells in the top 5% of fluorescence from the resveratrol-induced library (Supplementary Fig. 16). This subpopulation was grown as described previously and induced with 95 μM resveratrol at an OD_600_ of 0.2. The final sort consisted of isolating 500,000 cells from the bottom 60% of the nontreated fluorescence distribution. The sorted cells were incubated at 37 °C until the culture reached an OD_600_ of 0.2. A frozen stock was stored at −80 °C in 25% glycerol.

Aliquots of the sorted library, wild-type TtgR cotransformed with the reporter plasmid, and a GFP-positive control were thawed on ice. 50 μL of the library was plated on LB-kanamycin/spectinomycin and incubated at 37 °C for 16 h. The GFP control aliquot was streaked on LB-kanamycin and the wild-type TtgR aliquot was streaked on LB-kanamycin/spectinomycin and incubated in the same fashion. Colonies were selected from each plate and inoculated into 150 μL of LB in a 96 well plate. The colonies were incubated at 37 °C shaking in a SBT1500-H microplate shaker (Southwest Science) and grew to saturation (approximately 8 h). The cultures were diluted 15X into fresh LB with either 1000 μM naringenin or 100 μM resveratrol and incubated in a Synergy HTX plate reader (BioTek) for 16 h at 37 °C. The performance of each colony was measured using the ratio of fluorescence to optical density (RFU/OD_600_). The ratio of this measurement in the presence and absence of ligand defined the response to each ligand. Successful colonies had higher response for resveratrol than for naringenin. These colonies were sequenced using Sanger sequencing.

### Testing of combinatorial mutants

The 14 mutational intermediates were generated using eight primers specifically encoding combinations of either 137 + 141 or 167 + 168. The resulting oligos were inserted into the *TtgR*-pSC101 plasmid using isothermal assembly using .042pmol of backbone and 0.8pmol *TtgR*. DH10B *E.coli* cells (NEB) were transformed with the resulting reaction via electroporation. Colonies were selected and sequenced to verify the correct mutations were present. The correct colonies were inoculated into LB-spectinomycin and incubated at 37 °C for 16 h. An aliquot was stored at −80 °C in 25% glycerol and plasmids were harvested from the remaining culture. DH10B cells were cotransformed with the 14 *TtgR*-pSC101 plasmids and the pColE1 reporter plasmid. These were grown for 16 h shaking at 37 °C in LB-kanamycin/spectinomycin media and frozen in 25% glycerol at −80 °C.

A 250 mM stock of naringenin was made in DMSO and a 100 mM stock of resveratrol was made in ethanol. The *TtgR*-pSC101/pColE1 frozen stocks were struck out onto LB-kanamycin/spectinomycin plates. Colonies were selected and inoculated into 150uL LB in a 96-well plate. These grew in a microplate shaker to saturation (approximately 8 h) at 37 °C. The cultures were diluted 15X into fresh LB-kanamycin/spectinomycin in a 96-well plate with varying concentrations of either naringenin (0, 10, 25, 50, 75, 100, 250, 500, 750, 1000, 1500, 2000 μM) or resveratrol (0, 2.5, 5, 7.5, 10, 25, 50, 75, 100, 150, 200, 250 μM). The concentration series for each ligand differ due to solubility limits in aqueous solutions. A series of naringenin and resveratrol stock concentrations were made such that a 50X or a 100X dilution, respectively, would yield the desired concentrations in the assay. Most variants were assayed with three biological replicates. Variants whose standard deviation was greater than 10% of the mean fluorescence (1010, 1001, 1110, and 1101 for naringenin and 1001, 1000, 0001, and 0011 for resveratrol) were assayed with six replicates. The assay was incubated in the microplate shaker for 14 h at 37 °C shaking. Cells containing wild-type *TtgR*-pSC101 with the pColE1 reporter and cells containing pColE1 reporter alone served as controls and were included on every plate. A set of six biological replicates of a sfGFP positive control were induced with both sets of ligands and concentrations.

Cells were diluted 50X in ice-cold PBS. Fluorescence measurements were conducted on a LSR-Fortessa system (BD Biosciences) in the FACSDiva V8.0 software using a 488 nm laser for excitation and a 530/30 filter for fluorescence emission. Using gates on FSC-H vs FSC-A, 100,000 events were gathered per well (Supplementary Fig. 16). To account for changes in fluorescence that are independent of TtgR function, raw fluorescence values were normalized by fold changes in sfGFP fluorescence in the positive control (*N* = 6). The median values of the fluorescence distributions were used as the basis for fold induction calculations. Fold induction as calculated by obtaining the ratio of induced average median fluorescence to baseline average median fluorescence. In Fig. 1, fold induction values were calculated by obtaining the ratio of each biological replicate prior to averaging the ratios.

1 $${fold}\,{induction}=\frac{{F}_{{{max }}}}{{F}_{{baseline}}}$$

### Quantifying epistasis

The mean and standard deviation of each concentration of ligand for each combinatorial mutant were used to calculate a fit using the Hill equation as a function of ligand concentration (*x*)^43^:

2 $$f(x,n,E{C}_{50})={F}_{{baseline}}+\left(({F}_{{max}}-{F}_{{baseline}})* \left(\frac{{x}^{n}}{E{{C}_{50}}^{n}+{x}^{n}}\right)\right)$$

TtgR function was defined as the maximum fold induction of the system, which is the ratio of the median fluorescence at the highest ligand concentration and the median fluorescence at 0 μM ligand (Eq. 1). The Python 2.7 function curve_fit() from the Scipy module was used to fit the dose–response curves to the Hill equation (Supplementary Figs. 17,  18)^44^. This function provides both fit parameters and error as a covariance matrix as output. Basal gene expression was the fluorescence at 0 μM ligand. Maximum gene expression was the fluorescence at the highest ligand concentration. EC_50_ was estimated using the Hill equation (Eq. 2).

The Bahadur expansion was used to analyze the data^36^. Fitness for the bahadur expansion was defined as

3 $${{fitness}}_{{variant}}={log}_{10}\left(\frac{{fold}\,{{induction}}_{{variant}}}{{fold}\,{{induction}}_{{wildtype}}}\right)$$

Fold induction in Eq. (2)was changed to basal gene expression, maximum gene expression, or EC_50_ for each functional parameter. Each mutant can be represented as a numerical string (z string), where each mutable position is one number (*z*_*i*_) in the string. A wild-type residue at a position is designated by a −1 while the mutated residue is designated by a 1. The mutant M167L+F168Y thus becomes [−1, −1, 1, 1]. The interaction terms can be modeled as follows:

4 $${\varphi }_{0}=1$$

5 $${\varphi }_{1},{\varphi }_{2},\ldots ,{\varphi }_{n}={z}_{1},{z}_{2},\ldots ,{z}_{n}$$

6 $${\varphi }_{n+1},{\varphi }_{n+2},\ldots ,{\varphi }_{n+{C}_{2}^{n}}={z}_{1}{z}_{2},{z}_{1}{z}_{3},\ldots ,{z}_{n-1}{z}_{n}$$

7 $${\varphi }_{{2}^{n}-1}={z}_{1}{z}_{2}\ldots {z}_{n}$$

An orthonormal matrix of psi-values is created based on the combinations of mutations within the set (Supplementary Table 4). The Bahadur coefficients can be calculated using this orthonormal matrix and a fluorescence values *f*(*x*) for a particular mutant *x* in the set of all mutants X.

8 $${w}_{i}=\frac{1}{{2}^{n}}\mathop{\sum }\limits_{x\in X}f(x){\varphi }_{i}(x)$$

The fluorescence of each combinatorial mutant can be calculated based on the Bahadur coefficients and z string.

9 $$f(x)=\mathop{\sum }\limits_{i=0}^{2n-1}{w}_{i}{\varphi }_{i}(x)$$

The *R*^2^ between the modeled fluorescence values and the experimental data is 1.0 when all interaction terms are included in the expansion. By truncating Eq. (9) to contain only low-order interactions, the effect of these contributions to the model can be determined. The expansion was applied to the full set of mutations (4 positions) and modeled using first-order terms; first- and second-order terms; first-, second-, and third-order terms; and all terms (Supplementary Fig. 19). An identical approach was applied to all 24 subnetworks and utilized only first-order terms in the reconstruction (Supplementary Fig. 20).

Errors in the *R*^2^ statistics were estimated using a Monte Carlo simulation. 500 sets of fitness values for all mutants were sampled based on experimental fitness means and standard deviations following a Gaussian distribution using the NumPy module in Python 2.7^45,46^. Equations (8) and (9) were applied to reconstruct the fitness values and calculate *R*^2^ values between the sampled model and the sampled data to give a distribution of *R*^2^ values. Bias-corrected adjusted 95% confidence intervals were calculated by obtaining the average *R*^2^ of 10,000 bootstrap iterations of the Monte Carlo simulation *R*^2^. The bahadur expansion was applied to each functional parameter.

A control set of additive data was used to calculate the *R*^2^ of data showing no epistasis (Supplementary Table 2). This control set was analyzed using the same approach as the subnetwork workflow.

### Protein characterization

The *TtgR* gene for variants 0000, 1000, 0100, 1100, and 1111 were cloned into a pET31B vector downstream of the T7 promoter for lac-inducible transcription control using isothermal assembly with 0.18pmol backbone and 0.392pmol *TtgR*. *MBP* was amplified with primers to add a C-terminal His-tag and TEV site and inserted into the *TtgR*-pET31B vector upstream of *TtgR* to create a MBP-His-TtgR fusion with a TEV cleavage site between the His-tag and the TtgR protein. BL21 chemically competent cells (NEB) were transformed with 20 ng of pET31B vector. Dilutions of transformants were plated on LB-ampicillin agar. A colony was selected and grown in 5 mL LB-ampicillin media shaking at 37 °C for 16 h. This culture was added to 500 mL autoinduction media (Terrific Broth, 0.8% glycerol, 2 mM MgSO_4_, 0.375% (w/v) aspartic acid, 0.015% (w/v) glucose, 0.5% (w/v) lactose) and grown for 8 h at 37 °C shaking. The culture was grown for an additional 16 h at 25 °C shaking.

The cells were spun down at 5500 *g* for 15 min at 4 °C. The supernatant was removed and the cells were resuspended in a lysis buffer (300 mM NaCl, 50 mM HEPES, 1 mM PMSF, 1 mg/mL Lysozyme, 5 mM BME, 10% glycerol, pH 7.5). A Q500 sonicator (Qsonica) was used to lyse cells using a 5 s on, 15 s off sonication protocol for 4 min total sonication time. The lysate was centrifuged at 14,000 *g* for 45 min at 4 °C. The supernatant was isolated and filtered through a 0.22 μm filter. The filtered supernatant was purified on an Akta Start using 2 5 mL HisTrap HP columns. The column was washed with 5 column volumes (CV) IMAC-A (500 mM NaCl, 20 mM Imidazole, 20 mM MOPS, 0.3 mM TCEP, pH 7). MBP-6His-TtgR was eluted with a gradient of 100% IMAC-A to 100% IMAC-B (500 mM NaCl, 500 mM Imidazole, 20 mM MOPS, 0.3 mM TCEP, pH7) over 5CV and collected in 2 mL fractions. Fractions with the highest absorbance at 280 nm (A280) were combined and dialyzed in 8 L of dialysis buffer A (100 mM NaCl, 20 mM MOPS, 0.3 mM TCEP, pH 7.5). TEV was added to the proteins prior to dialysis at a ratio of 1:50 w/w TEV:TtgR. Dialysis occurred over a 16 h interval at 4 °C while stirring at low speed.

Dialyzed protein was centrifuged at 14,000 *g* for 10 min at 4 °C. The supernatant was passed through a 0.22 μm filter and loaded onto the HisTrap columns at 5 mL/min. The column was washed with 5CV of IMAC-A and 2 mL fractions were collected. 5CV of IMAC-B was used to remove the MBP-6His from the column. The column was washed with an additional 10CV IMAC-A. Wash fractions with high A280 were combined and reapplied to the column. The column was washed with 5CV of IMAC-A and 2 mL fractions were collected. 5CV of IMAC-B was used to strip the MBP-6His from the column. Fractions with high A280 were combined and dialyzed in 4 L of dialysis buffer C (100 mM NaCl, 20 mM MOPS, 10 mM MgCl_2_, 0.3 mM TCEP, pH 7.8). The protein was centrifuged at 14,000 *g* for 10 min at 4 °C. The supernatant was passed through a 0.22 μm filter. The protein was concentrated to approximately 9 mg/mL and frozen in 60 μL aliquots in liquid nitrogen before storing at −80 °C. Dialysis buffer C was passed through a 0.22 μm filter and stored at 4 °C for ITC experiments.

Stocks of 250 mM naringenin and 100 mM resveratrol were diluted to 500 μM and 250 μM, respectively, in dialysis buffer C. Aliquots of TtgR were thawed on ice and diluted to a final concentration of 7.5 μM. DMSO or ethanol was added to the TtgR solution to match the solution composition of the naringenin or resveratrol dilutions. An aliquot of dialysis buffer C was also prepared with DMSO or ethanol for a control injection and to wash the sample cell between ITC injections.

The ITC experiments were conducted on a VP-ITC (MicroCal). An initial control injection scheme consisted of loading the sample cell with dialysis buffer C and performing a series of 10 10 μL ligand injections with 10 min intervals at 25 °C. The sample cell was washed 5 times with dialysis buffer C before the 7.5 μM protein solution was loaded. Twenty-five 10 μL naringenin injections or 28 10 μL resveratrol injections occurred in 10 min intervals at 25 °C.

Data analysis was primarily conducted using Origin 7.0 (MicroCal). The heats of injection from the control sample were averaged. The protein-ligand injection profile was subtracted by this average heat prior to curve fitting. Due to low affinity for both naringenin and resveratrol, the stoichiometry of binding was fixed to 1 to reduce the degrees of freedom prior to fitting. The curves were fit with the single binding site model (Supplementary Fig. 21).

### X-ray crystallography

*TtgR*-pET31B vector was electroporated into BL21 cells (NEB) and recovered in 1 mL SOC. The cells were incubated for 1 h at 37 °C before serial dilutions were plated on LB-ampicillin (100 μg/mL) plates. A single colony was selected and incubated in 5 mL LB-ampicillin (100 μg/mL) at 37 °C shaking for 3 h. The 5 mL culture was added to 500 mL LB-ampicillin media and incubated at 37 °C shaking at 250 rpm for approximately 3 h until the OD_600_ reached 0.6. The culture was induced with 100 μM IPTG followed by an incubation at 16 °C for 16 h shaking at 250 rpm.

The cells were spun down at 5500 *g* for 15 min at 4 C. The supernatant was removed and the cells were resuspended in a lysis buffer (300 mM NaCl, 50 mM HEPES, 1 mM PMSF, 1 mg/mL Lysozyme, 5 mM BME, 10% glycerol, pH 7.5). A Q500 sonicator (Qsonica) was used to lyse cells using a 25 s on, 50 s off sonication protocol for 3 min and 45 s total sonication time. The lysate was centrifuged at 14,000 *g* for 45 min at 4 °C. The supernatant was isolated and filtered through a 0.22 μm filter. The filtered supernatant was purified on an Akta Start (Cytiva) using 5 mL HisTrap HP columns (Cytiva). The supernatant was loaded onto the column at a flow rate of 5 mL/min. The column was washed with 5 column volumes (CV) IMAC-A. MBP-6His-TtgR was eluted with a gradient of 100% IMAC-A to 100% IMAC-B over 10CV and collected in 2 mL fractions. Fractions with the highest absorbance at 280 nm (A280) were combined and dialyzed in 8 L of dialysis buffer A. TEV was added to the proteins prior to dialysis at a ratio of 1:50 w/w TEV:TtgR. Dialysis occurred over a 16 h interval at 4 °C while stirring at low speed.

TtgR was isolated from MBP-6His through a subtractive IMAC protocol using the Akta Start and 5 mL HisTrap HP column. The dialyzed protein was centrifuged at 4000 *g* for 10 min at 4 C. Supernatant was passed through a 0.22 μm filter and applied to the HisTrap column at 5 mL/min. 5CV IMAC-A was used to wash the column while 2 mL fractions were collected. 2.5CV IMAC-B was used to remove the MBP from the column and 5 mL fractions were collected. Wash fractions with high A280 were combined and dialyzed in 4 L of dialysis buffer B (50 mM NaCl, 5 mM MOPS, 0.3 mM TCEP, pH 7.5). EDTA was added to the protein wash fractions to a final concentration of 10 mM prior to dialysis. Dialysis occurred over a 16 h interval at 4 C while stirring at low speed. TtgR was concentrated to 10 mg/mL using spin concentrators. Samples were spun at intervals of 3500 *g* for 5 min and mixed via pipette between spins. Concentrated TtgR was separated into 60 μL aliquots and frozen in liquid nitrogen prior to storage at −80 °C.

Samples of TtgR wild type and mutant proteins were received frozen in 5 mM MOPS, pH 7.4, 50 mM NaCl, 0.3 mM TCEP. Samples were thawed and centrifuged for 5 min at 21,130 *g*. Sample supernatants were filtered with a 0.22 micron MillexGV syringe filter unit (Millipore) before applying to an equilibrated 10 mm × 300 mm Superdex 200 column (GE Healthcare). Chromatography was performed on a GE AKTA FPLC system. Column buffer was 20 mM HEPES, pH 7.5, 350 mM NaCl, 0.3 mM TCEP. Two primary peaks were obtained from each sample with major peak at approximately 45kD MW and a minor peak at approximately 79kD. The fractions corresponding to the major peak were pooled and concentrated with an Amicon Ultracel-10 centrifugal filter device (Millipore) and dialyzed vs. 5 mM HEPES, pH 7.5, 50 mM NaCl, 0.3 mM TCEP. Samples collected after dialysis were divided into small aliquots and flash frozen in PCR tubes with liquid nitrogen.

Crystallization screening and optimization were conducted in the Collaborative Crystallography Core in the Department of Biochemistry and the University of Wisconsin-Madison. Crystallization experiments were set up using a SPT Labtech mosquito® crystallization robot in MRC SD-2 crystallization plates at 4 °C and 20 °C (277 and 293 K.) Crystals progressing to diffraction experiments were all obtained at 20 °C. Two general screens, Hampton Research IndexHT and Molecular Dimensions JCSG+ were used in this study^47^. Crystals were detected using brightfield and UV fluorescence imaging with a JANSi UVEX-P crystallization plate imaging system supplementing visual inspection with stereomicroscopes. Initial rounds of crystallization optimization were performed in SD2 plates using the mosquito to expand 24 solution conditions by setting columns of experiments in four different samples to reservoir volume ratios. Cryoprotected crystals were harvested in Mitegen micro mounts and flash cooled by immersion in liquid nitrogen.

Crystals were screened and X-ray diffraction data were collected at Advanced Photon Source (APS) beamlines LS-CAT and GM/CA@APS, universally on crystals cooled to 100 K. Diffraction data was reduced using XDS (VERSION Mar 15, 2019 BUILT=20190315) and scaled with XSCALE (VERSION Mar 15, 2019 BUILT=20190315)^48,49^. Structures were solved by molecular replacement with Phaser V2.8.2 within the Phenix suite of programs (V1.18.2_3874), automatically rebuilt with phenix.autobuild, iteratively improved with alternating rounds of rebuilding in Coot and refinement using phenix.refine, and validated using MOLPROBITY V4.02-528^50–54^.

### 7K1A

Crystals providing diffraction data were grown by mixing 200 nL of protein at 9.7 mg/mL in sample buffer (5 mM HEPES pH 7.5, 50 mM NaCl, 0.3 mM TCEP) with 150 nL of reservoir solution, was equilibrated against 150 nL 20% MEPEG, 0.2 M MgCl_2_, 0.1 M bistris HCl pH 6.5 equilibrated against 50 μL of reservoir solution in a SD2 plate. Samples were cryoprotected with reservoir solution supplemented to 35% MEPEG 2000. A 360° sweep of data (720 frames) was collected on a MAR 300 CCD detector at LS-CAT beamline 21ID-G on 2018-12-16 using 0.97856 Å X-rays. The phase problem was solved using 2UXU(A) as a molecular replacement model^28^.

### 7K1C

Crystals of wild-type TtgR with resveratrol were prepared by incubating 0.41 mM protein (9.8 mg/mL) and 0.5 mM resveratrol dissolved in sample buffer for 30 min at room temperature prior to setting up crystallization experiments. The crystal yielding the best diffraction data were grown by mixing 200 nL of the protein-ligand sample with 250 nL reservoir (18% PEG4000, 0.2 M MgCl_2_, 0.1 M bistris HCl pH 6.5) equilibrated against 50 μL of reservoir a SD2 plate. Samples were cryoprotected with reservoir solution supplemented with 35% PEG4000. A 360° sweep of data (720 frames) was collected on a MAR 300 CCD detector at LS-CAT beamline 21ID-G on 2018-12-16 using 0.97856 Å X-rays. The phase problem was solved using 2XDN as a molecular replacement model.

### 7KD8

Crystals were prepared by incubating 0.43 mM (10.4 mg/mL) quadruple mutant protein with 1 mM resveratrol in sample buffer for 30 min prior to setting up crystallization experiments. Crystals providing the reported diffraction data set grew from 2 μL of sample mixed with 2  μL of reservoir solution (12% MEPEG 2000, 5% 2-methyl-2,4-pentanediol, 0.3 M MgCl_2_, 0.1 M bistris buffer at pH 6.5 equilibrated in a hanging drop experiment using a siliconized glass cover slip. Samples were cryoprotected with reservoir solution supplemented to 30% MEPEG 2000. A 360°(3600 frames) shutterless data set was collected at LS-CAT 21ID-D on 2019-05-30 with an Eiger 9 M direct detector and 1.07812 Å X-rays. The phase problem was solved using 7K1A as a molecular replacement model.

### Reporting summary

Further information on research design is available in the Nature Research Reporting Summary linked to this article.

## Supplementary information

Supplementary Information

Reporting summary

## Data Availability

The datasets generated and analyzed during the current study are available from the corresponding author on reasonable request. The crystallography data generated in this study are available in the RCSB under the following accession codes: 7K1A, 7K1C, and 7KD8. The naringenin-bound wild-type TtgR structure data used in this study are available in the RCSB Protein Data Bank under accession code 2UXU. The minocycline-bound TtgR structure data used for computational design are available in the RCSB Protein Data Bank under accession code 2UXH. Source data are provided with this paper.

## Source data

Source Data

## Code availability

All figures were generated using the Matplotlib module in Python 2.7^55^. Python and shell scripts for generating input from Rosetta and analyzing from Rosetta are available at: https://github.com/raman-lab/biosensor_design. Scripts used in data analysis of flow cytometry data, epistasis analysis, and figure generation can be found at: https://github.com/raman-lab/epistasis. Flow cytometry data were analyzed in FlowJo V10. ITC data were analyzed using Origin V7.0. POVME V3.0 was used to calculate pocket volumes based on the location of resveratrol^56^.
