# Supplementary material for: Epistasis shapes the fitness landscape of an allosteric specificity switch
Source: Nat Commun. 2021 Sep 21;12:5562. doi: 10.1038/s41467-021-25826-7 (PMC8455584; doi:10.1038/s41467-021-25826-7)
Supplement: Supplementary file 1 — Supplementary Information [file 41467_2021_25826_MOESM1_ESM.pdf]

Epistasis shapes the fitness landscape of an allosteric specificity switch

Supplementary Information

Kyle K. Nishikawa[1], Nicholas Hoppe[1], Robert Smith[1], Craig Bingman[1]

and Srivatsan Raman[1,2,3,§]

1 Department of Biochemistry, University of Wisconsin-Madison, Madison, WI
2 Department of Bacteriology, University of Wisconsin-Madison, Madison, WI
3 Department of Chemical and Biological Engineering, University of Wisconsin-Madison, Madison, WI

[§] Corresponding author: sraman4@wisc.edu

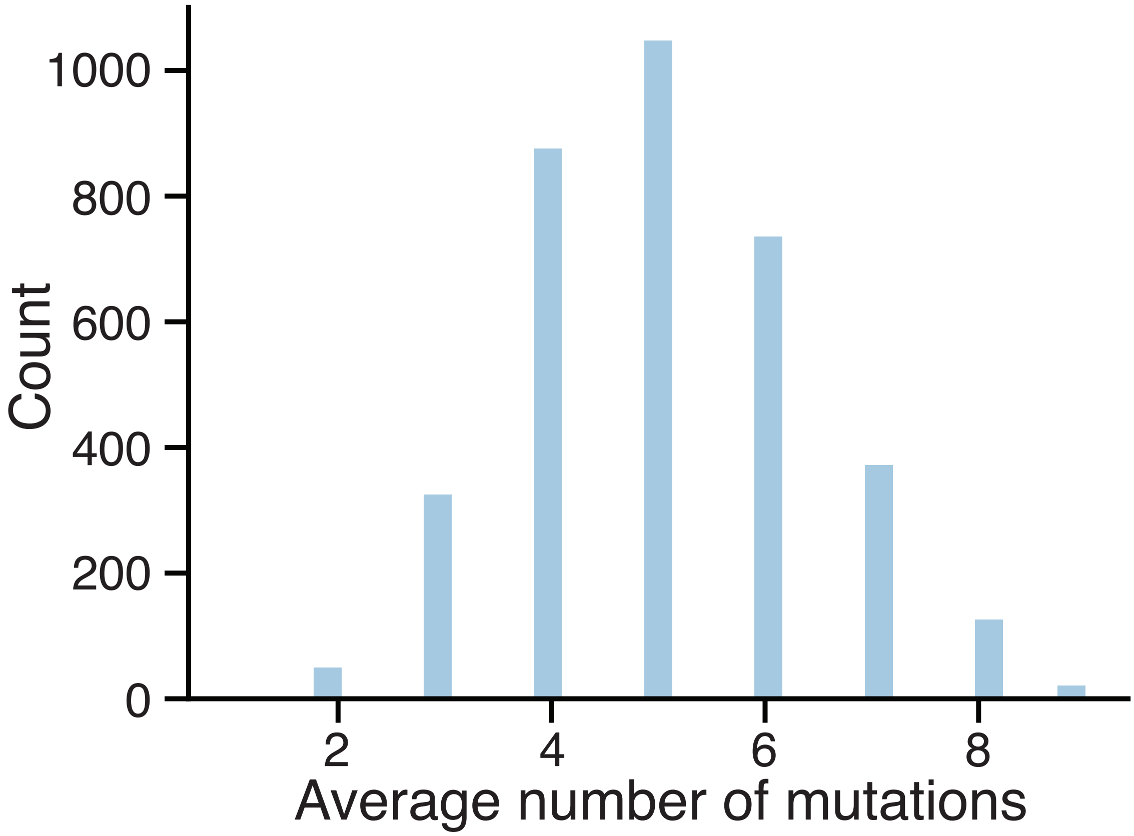

Supplementary Figure 1: Mutation distribution for synthesized resveratrol designs

Histogram of the number of mutations in the library of experimentally screened, Rosetta-generated designs. The average number of mutations was 5.1 with a variance of 1.8.

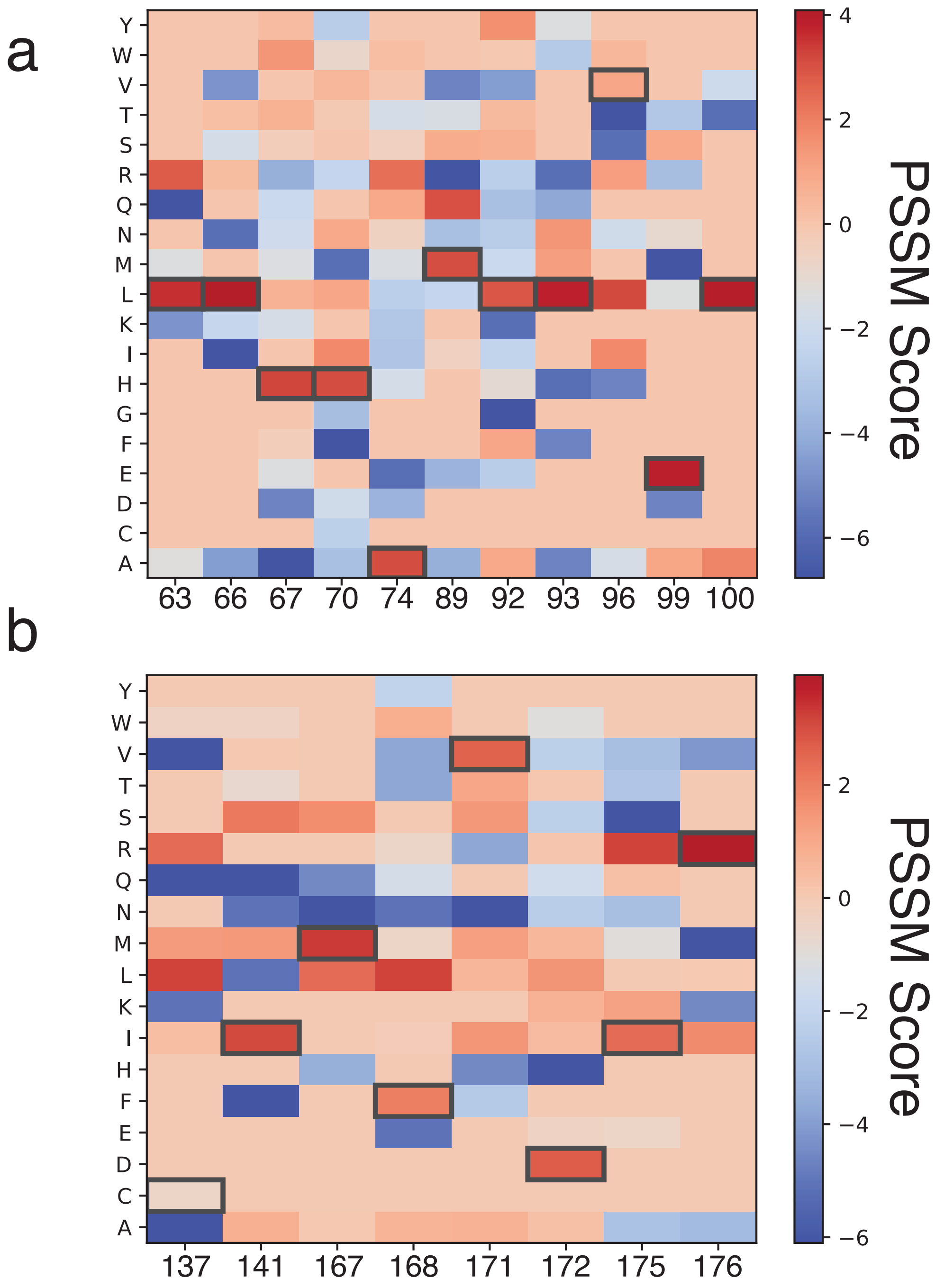

Supplementary Figure 2: Mutation heatmap for synthesized resveratrol designs
Heatmaps are colored by PSSM score calculated from the set of curated Rosetta designs. A black box is drawn around the wildtype amino acid identity at each position. **(a)** Heatmap of the first of two designed regions of TtgR. **(b)** Heatmap of the second designed region in TtgR.

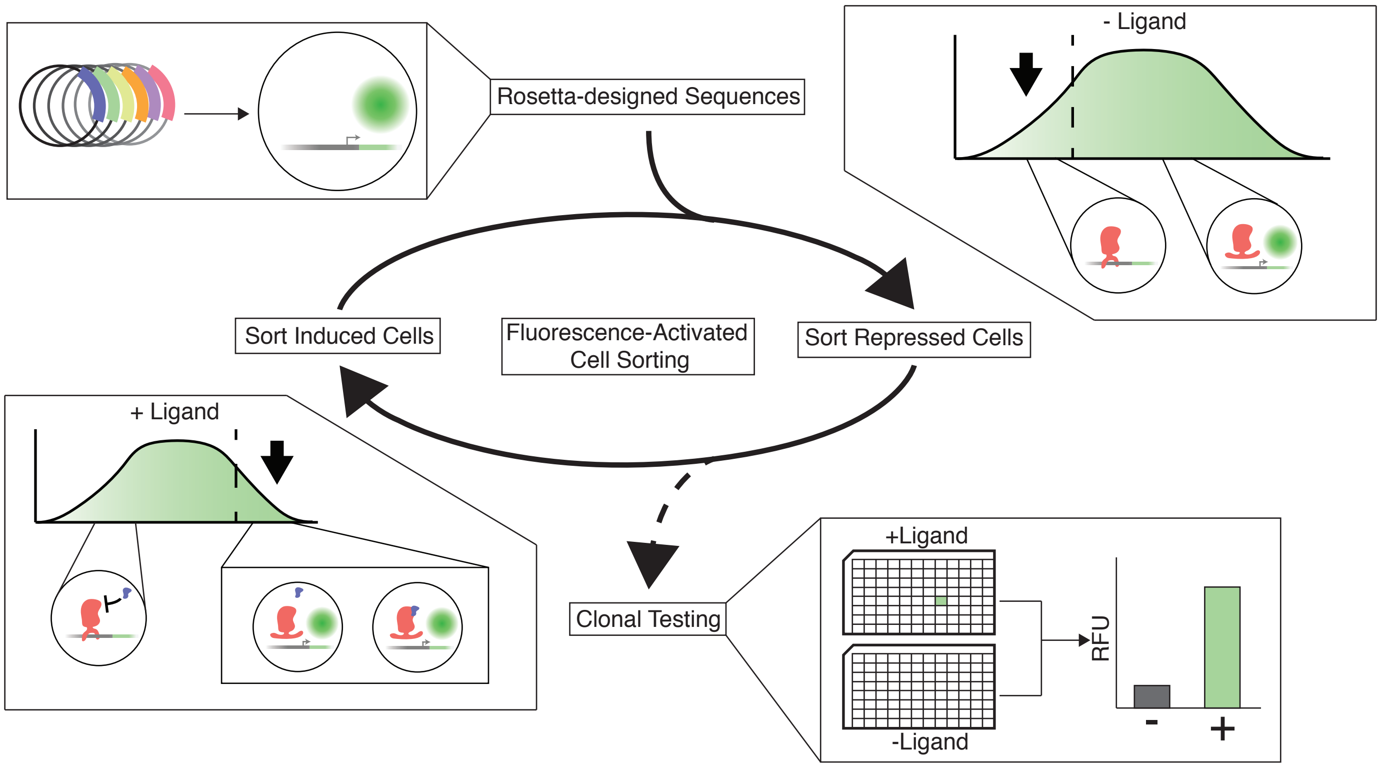

Supplementary Figure 3: Workflow for screening ligand-specific TtgR variants by fluorescence activated cell sorting

Rosetta-designed TtgR variants are transformed into *E. coli* cells carrying the reporter plasmid. TtgR variants are sorted by toggling between repressed and induced states (solid arrow). The lower 50% of fluorescent cells are sorted in the absence of inducer to isolate variants that are able to repress transcription. Subsequently, the sorted population are grown and induced with resveratrol. The top 5% of fluorescent cells are sorted to isolate variants capable of binding to the ligand and inducing GFP expression. After toggling multiple times, the repressed sort is repeated a final time before the subpopulation is clonally tested with both naringenin and resveratrol (dashed arrow).

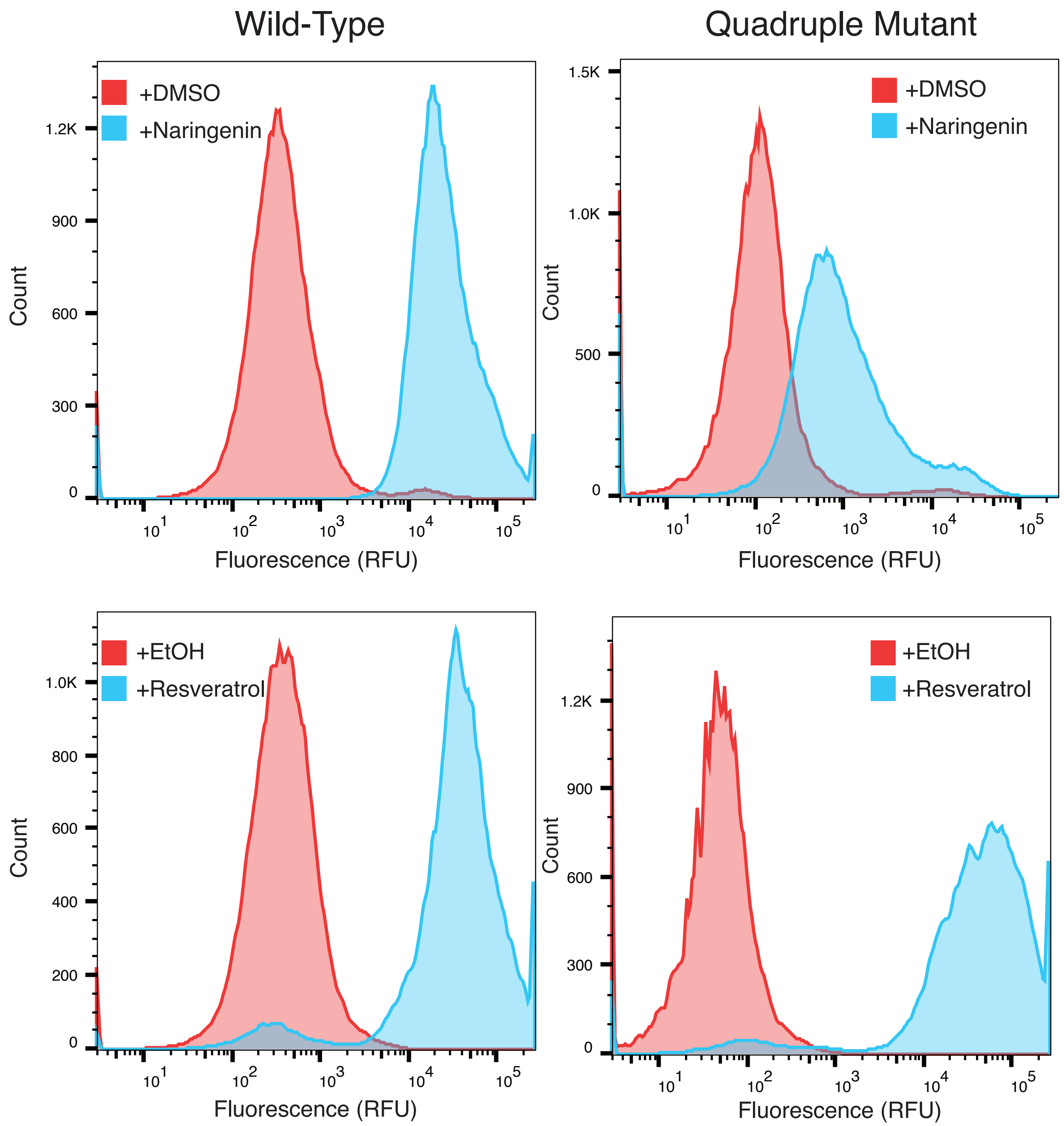

Supplementary Figure 4: Fluorescence distributions of wildtype TtgR and quadruple mutant

Flow cytometry histograms of wildtype TtgR and quadruple mutant TtgR with and without inducers. Naringenin 2000µM (blue) dissolved in DMSO and DMSO-only control (red), Resveratrol 250µM (blue) dissolved in ethanol and ethanol-only control (red) are shown.

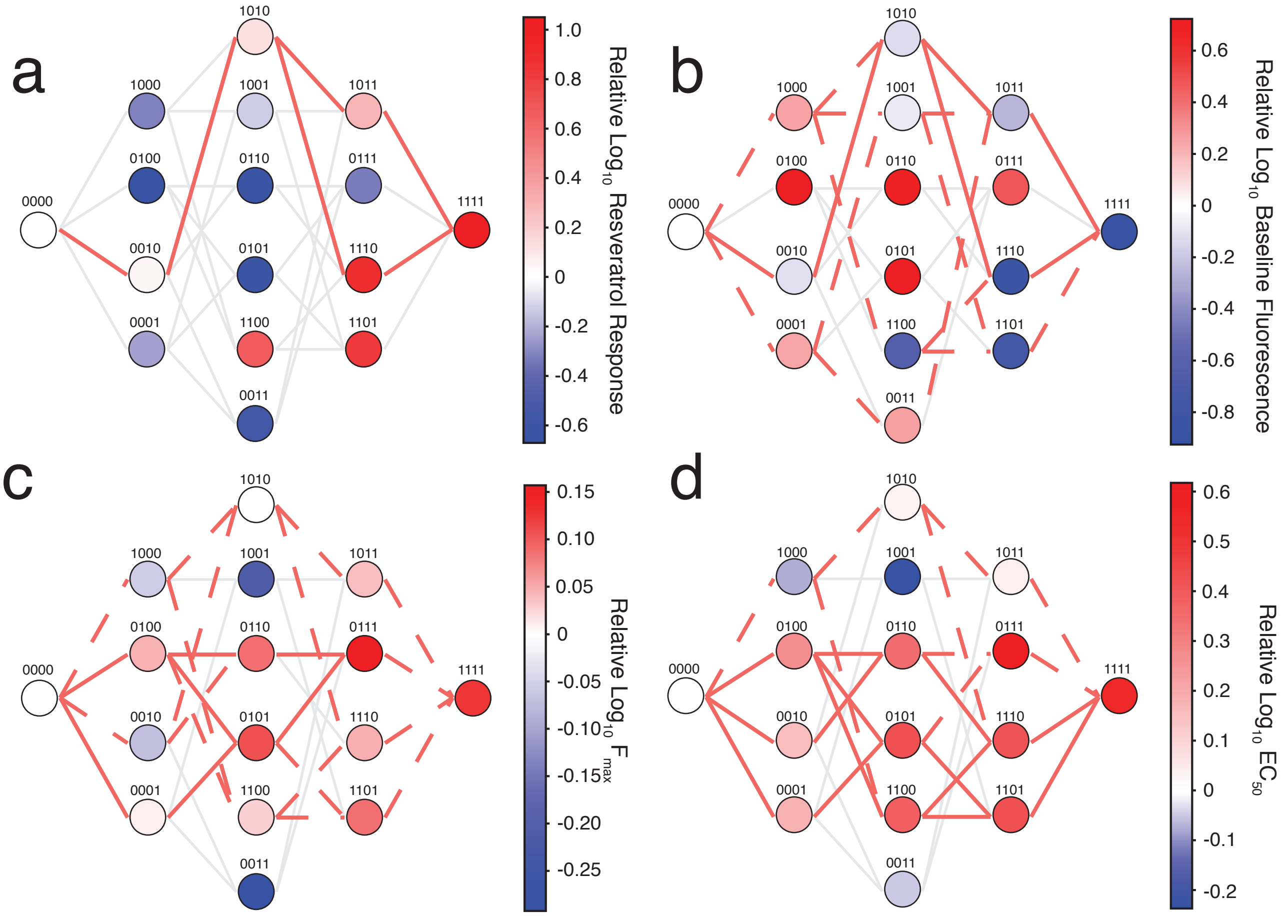

Supplementary Figure 5: Additional mutational pathways permitted by a 25% tolerance window for resveratrol functional parameters

The tolerance window describes the acceptance of a mutation that performs worse than the background variant when describing allowed pathways through sequence space. Each variant is labeled with a binary string corresponding to the presence (1) or absence (0) of a mutation at position 137, 141, 167, or 168 in order. Nodes separated by a single mutation are connected by edges showing viable (bold red) and unviable paths (light gray) through sequence space. Nodes are shaded by $\log_{10}$ of the fitness parameter at 250μM resveratrol normalized to the fitness of wildtype TtgR. All new tolerated pathways are shown as red dashed lines. Additional pathways have been calculated for resveratrol **(a)** fold induction, **(b)** basal expression, **(c)** maximum expression, and **(d)** EC_{50} landscapes. The fold induction landscape shows no additional pathways while the basal expression, maximum fluorescence, and EC_{50} landscapes show 11, 8, and 8 additional paths, respectively.

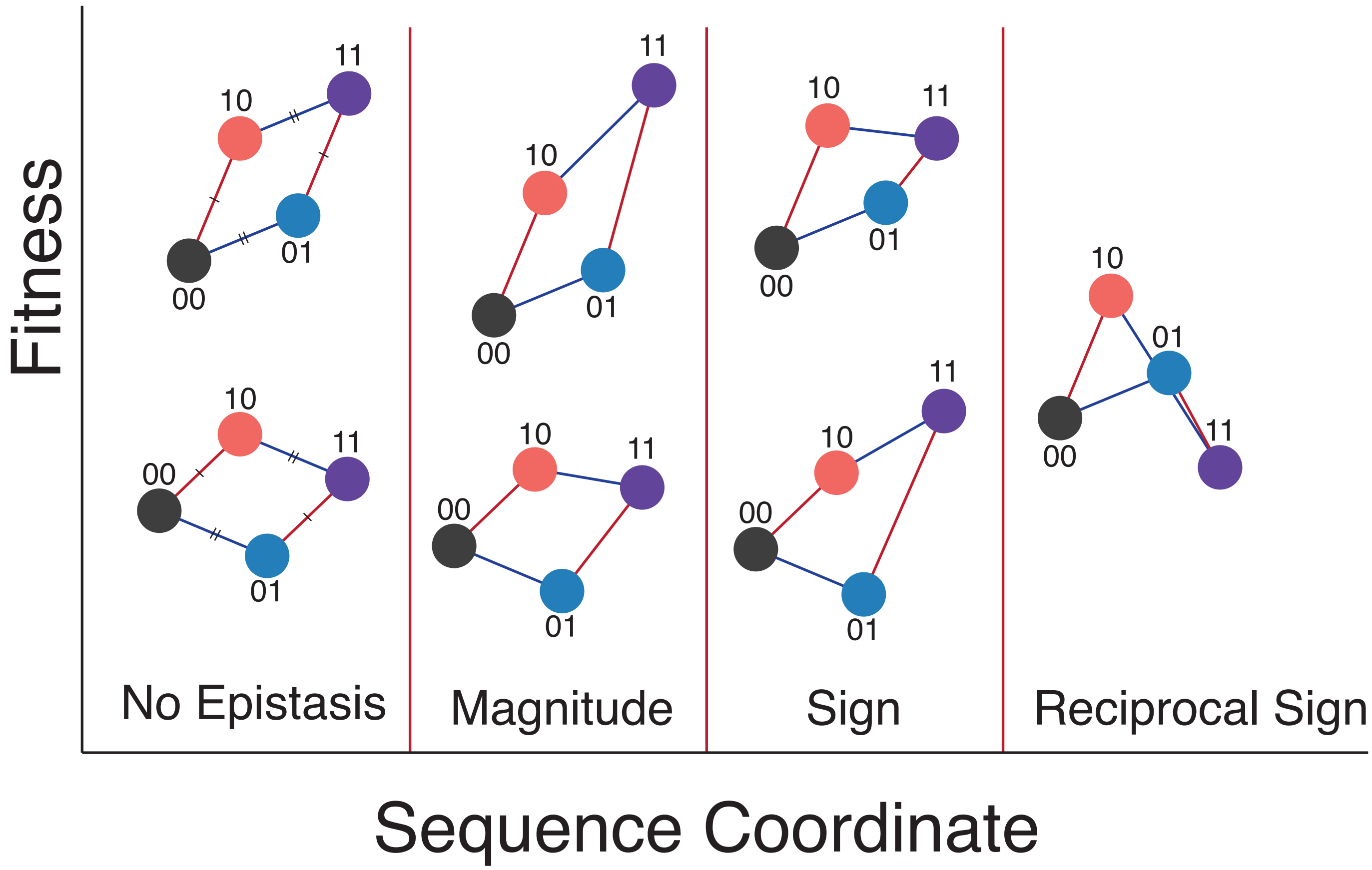

Supplementary Figure 6: Visual definition of different types of epistasis
Visual representation of different types of epistasis. This graphical representation separates example subnetworks based on the type of epistasis. An arbitrary fitness metric is plotted against a sequence coordinate where each mutation is represented by a binary string. A system is non-epistatic when the combined effect of mutations is the sum of their individual effects. Magnitude epistasis occurs when the combined effect of mutations is greater than the sum of their individual effects (no change in direction). Sign epistasis occurs when one mutation switches direction from beneficial to detrimental (or vice versa) depending on the background in which it is introduced. Reciprocal sign epistasis occurs when both mutations switch direction depending on the background.

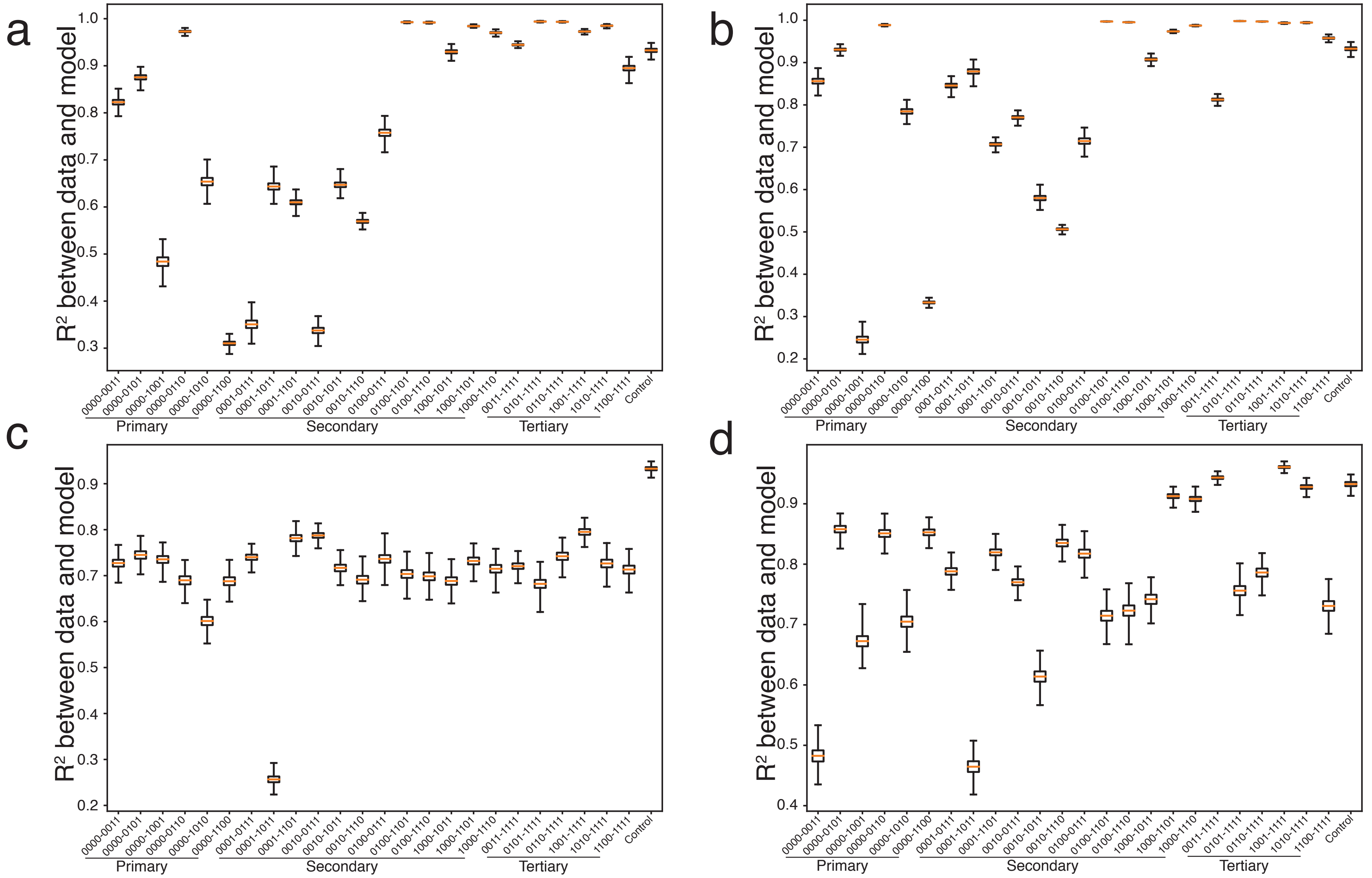

Supplementary Figure 7: Bahadur expansion of subnetworks in resveratrol functional parameters
Bahadur expansion was applied to the 24 subnetworks of the **(a)** fold induction, **(b)** basal expression, **(c)** maximum expression, and **(d)** EC_{50} landscapes. The box plots show the bootstrap averages (N=10,000 bootstrap replicates). Epistatic subnetworks were defined as those with an R^2 value of less than 0.9, based on simulated additive data ("Control"). The box denotes the interquartile range and the orange line denotes the median R^2 value for the bootstrap averages. The whiskers extend to the maximum and minimum R^2 values. The fold induction landscape shows that the majority of subnetworks in a wildtype or single-mutant background show epistasis while those in double-mutant backgrounds are less likely to show epistasis. The basal expression landscape shows similar patterns of R^2 values as the fold induction landscape. All of the subnetworks in the maximum expression landscape are epistatic. The EC_{50} landscape shows more epistasis than the fold induction landscape in subnetworks with a double mutant background.

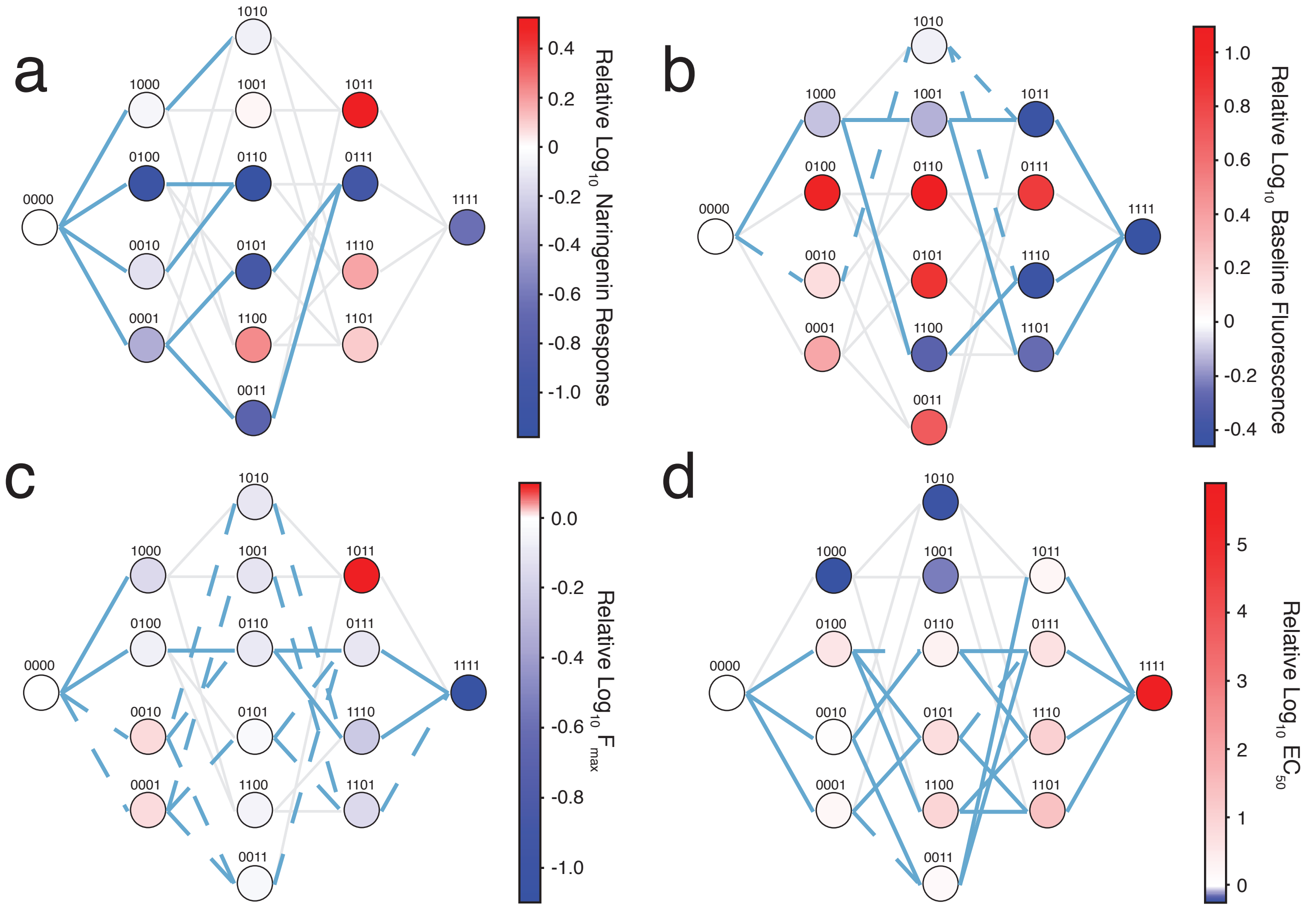

Supplementary Figure 8: Additional mutational pathways permitted by a 25% tolerance window for naringenin functional parameters
The tolerance window describes the acceptance of a mutation that performs worse than the background variant when describing allowed pathways through sequence space. Each variant is labeled with a binary string corresponding to the presence (1) or absence (0) of a mutation at position 137, 141, 167, or 168 in order. Nodes separated by a single mutation are connected by edges showing viable (bold red) and unviable paths (light gray) through sequence space. Nodes are shaded by $\log_{10}$ of the fitness parameter at 2000μM naringenin normalized to the fitness of wildtype TtgR. All new tolerated pathways are shown as blue dashed lines. Additional pathways have been calculated for resveratrol **(a)** fold induction, **(b)** basal expression, **(c)** maximum expression, and **(d)** EC_{50} landscapes. The fold induction landscape shows no additional pathways while the basal expression, maximum fluorescence, and EC_{50} landscapes show 2, 8, and 6 additional paths, respectively.

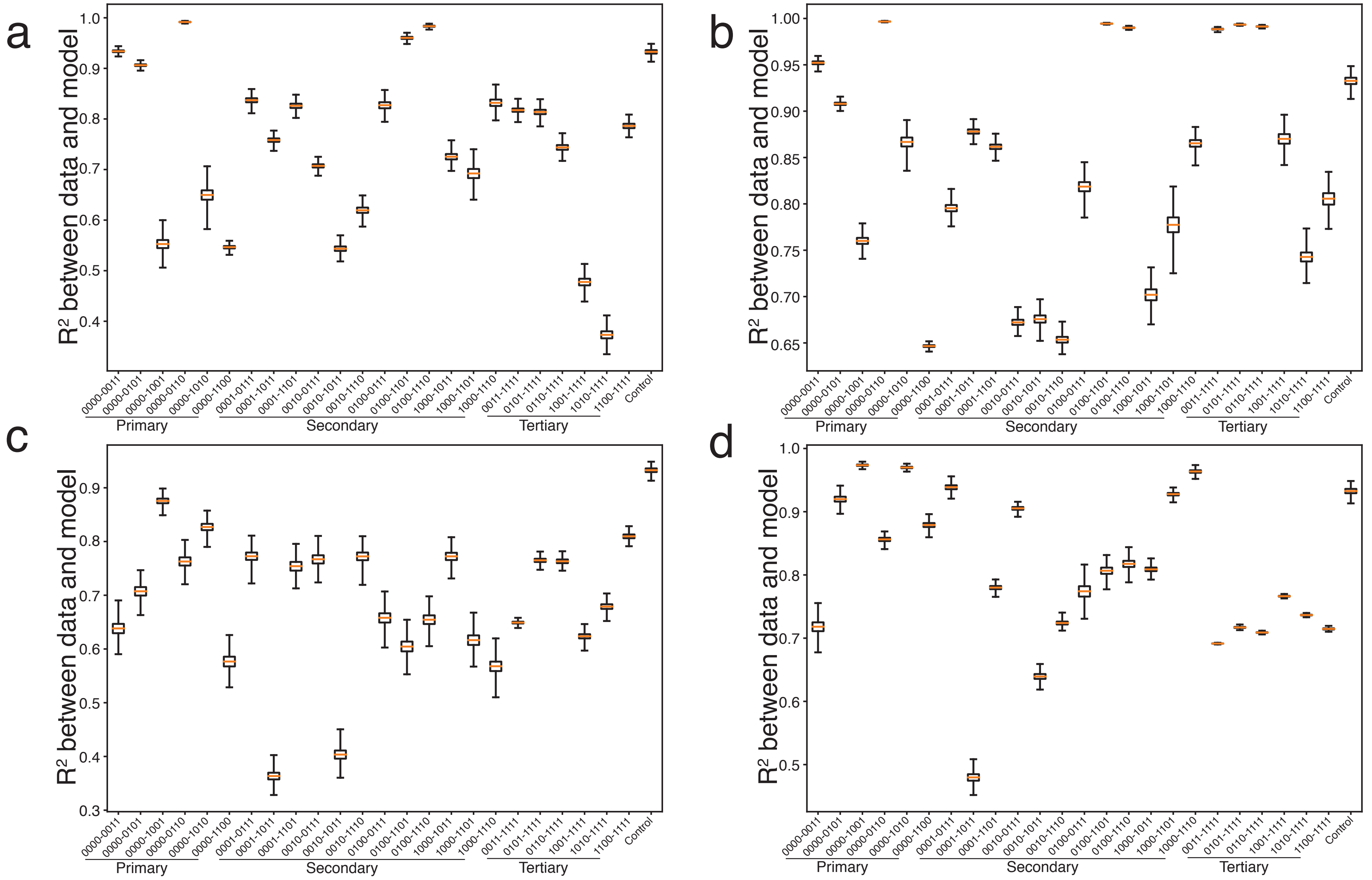

Supplementary Figure 9: Bahadur expansion of subnetworks in naringenin functional parameters

Bahadur expansion was applied to the 24 subnetworks of the **(a)** fold induction, **(b)** basal expression, **(c)** maximum expression, and **(d)** EC_{50} landscapes. Epistatic subnetworks are defined in the same fashion as Supplementary Fig. 7. The box plots show the bootstrap averages (N=10,000 bootstrap replicates). The box denotes the interquartile range and the orange line denotes the median R^2 value for the subnetwork. The whiskers extend to the maximum and minimum R^2 values. The fold induction landscape shows that only a small number of subnetworks in the wildtype or single mutant background are not epistatic. In contrast, the basal expression landscape has nonepistatic subnetworks in the wildtype, single, and double mutant backgrounds. Like the subnetworks of the resveratrol maximum fluorescence landscape, the majority of subnetworks in the naringenin maximum fluorescence landscape show epistasis. The EC_{50} landscape shows similarity to the fold induction landscape with nonepistatic subnetworks in the wildtype and single mutant backgrounds.

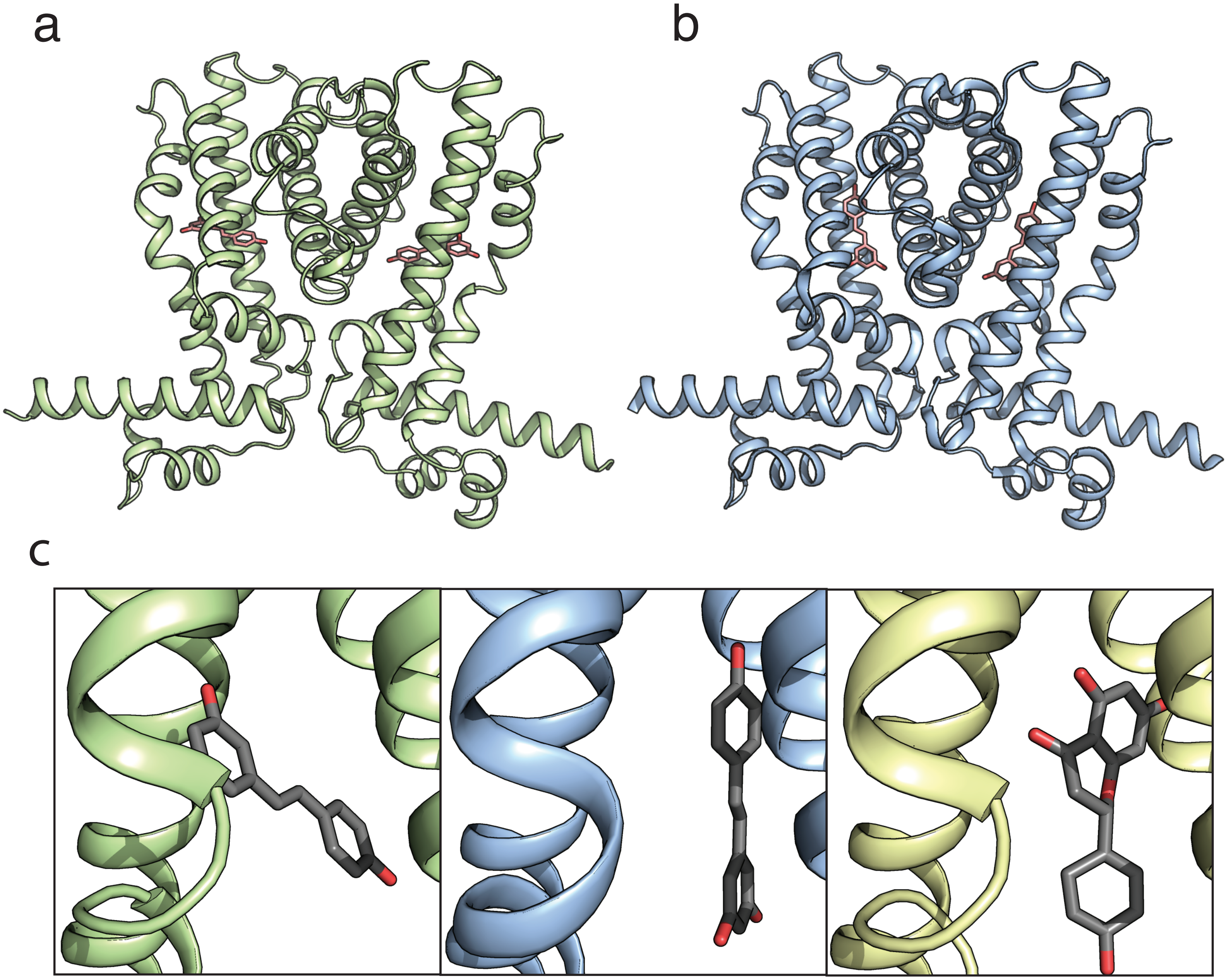

Supplementary Figure 10: Structure of ligand-bound wildtype and quadruple mutant TtgR is as an all-helical dimer. The helix-turn-helix domain at the N-terminal end binds to DNA. The ligand binding pocket is enclosed by five angled helices. An additional helix at the C-terminal end forms the dimerization interface. The quadruple mutant (PDB: 7KD8) **(a)** is structurally identical to the wildtype (PDB: 7K1C) **(b)**. Resveratrol is shown as pink sticks in both. **(c)** A close-up view of the binding orientation of resveratrol in the pocket. The quadruple mutant (left) binds to resveratrol in the horizontal orientation. Wildtype TtgR binds to resveratrol (middle) or to naringenin (right, PDB ID: 2UXU) in the vertical orientation. Resveratrol is shown as grey sticks.

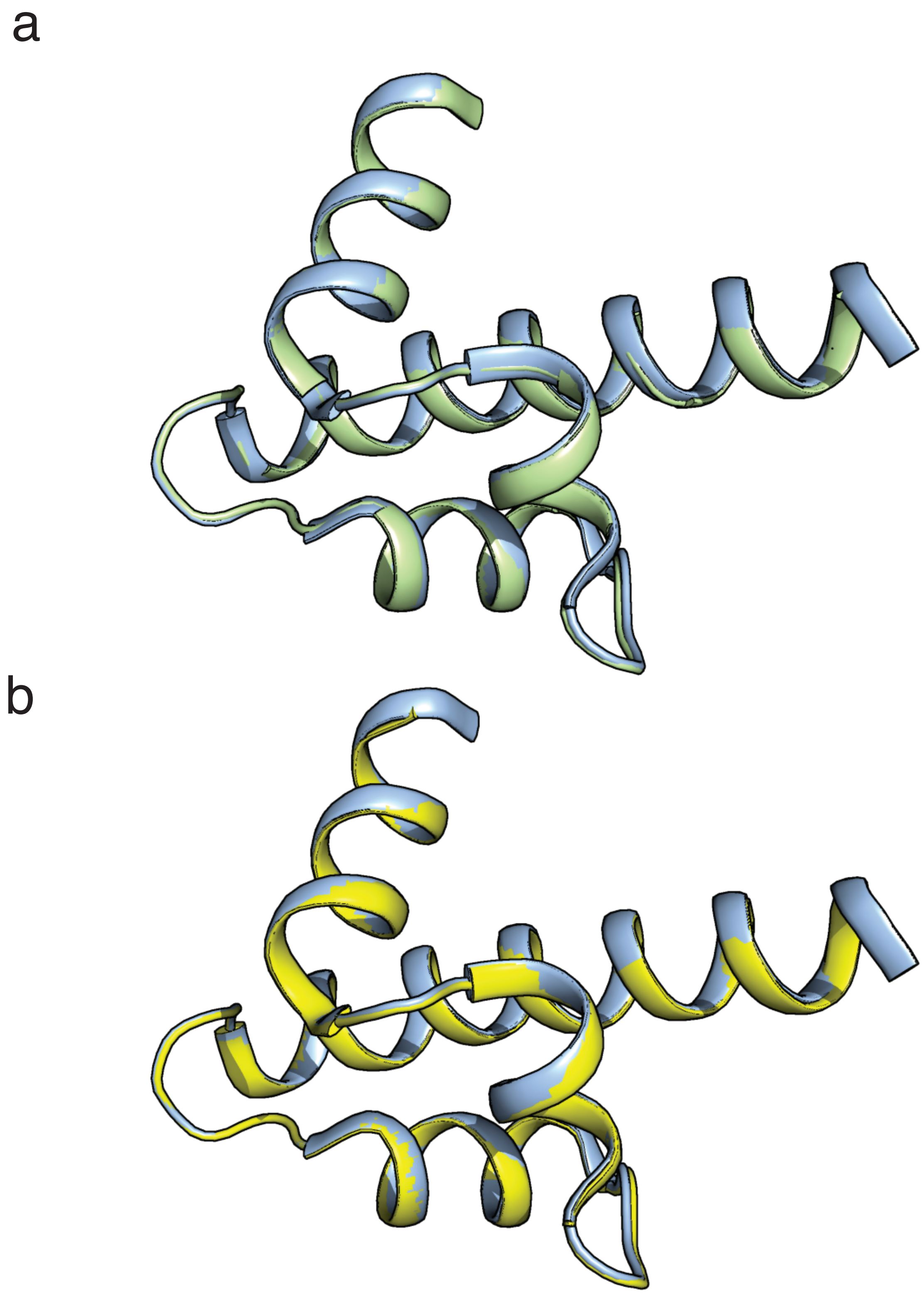

Supplementary Figure 11: Alignment of DNA binding domains for resveratrol-bound quadruple mutant, resveratrol-bound wildtype, and apo quadruple mutant TtgR
(a) The DNA binding domains of the resveratrol-bound quadruple mutant (green) and the resveratrol-bound wildtype (blue) TtgR. The RMSD of these two domains is 1.03Å. **(b)** The DNA binding domains of resveratrol-bound wildtype (blue) and apo wildtype (yellow) TtgR. The RMSD of these two domains is 1.35Å.

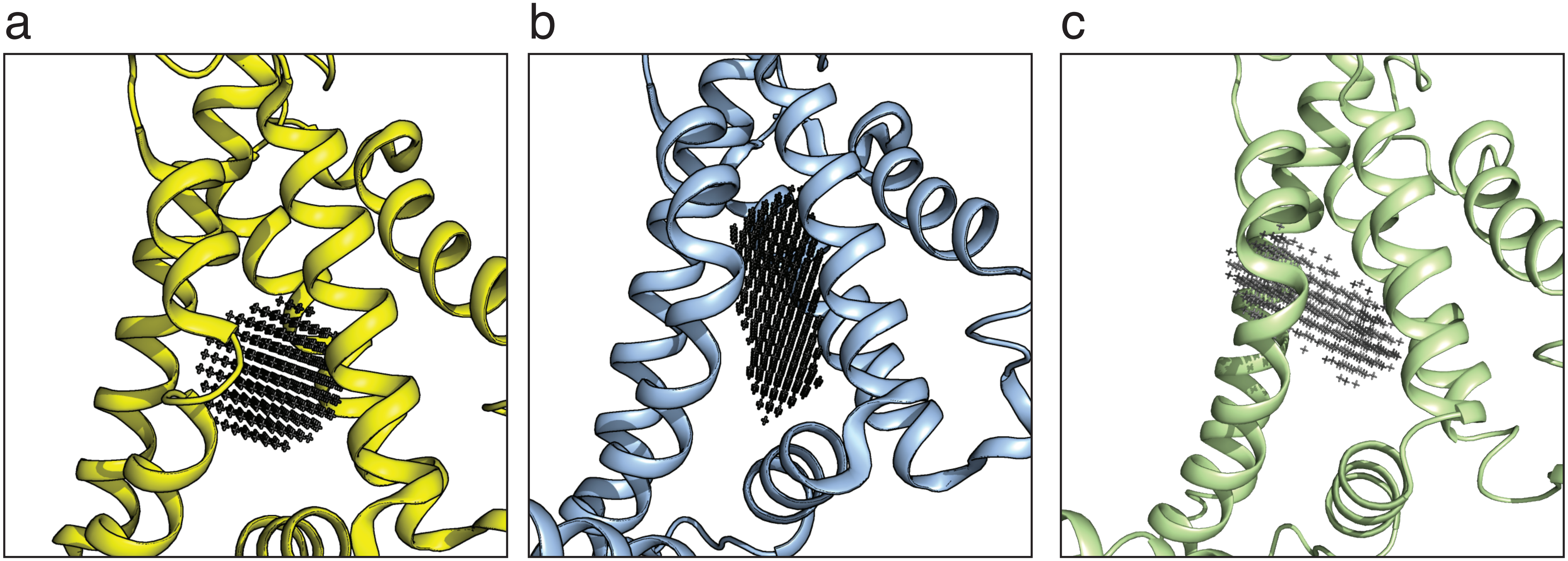

Supplementary Figure 12: TtgR binding pocket volume visualization
Binding pocket volumes were calculated using POVME3.0 and visualized in Pymol. **(a)** The pocket volume of apo wildtype TtgR, represented by the Xs, is 170Å^3. The pocket was not predefined in this calculation. **(b)** The pocket volume of resveratrol-bound wildtype TtgR is 215Å^3. **(c)** The pocket volume of resveratrol-bound quadruple mutant TtgR is 234Å^3. The pockets for (b) and (c) were defined using the resveratrol molecule.

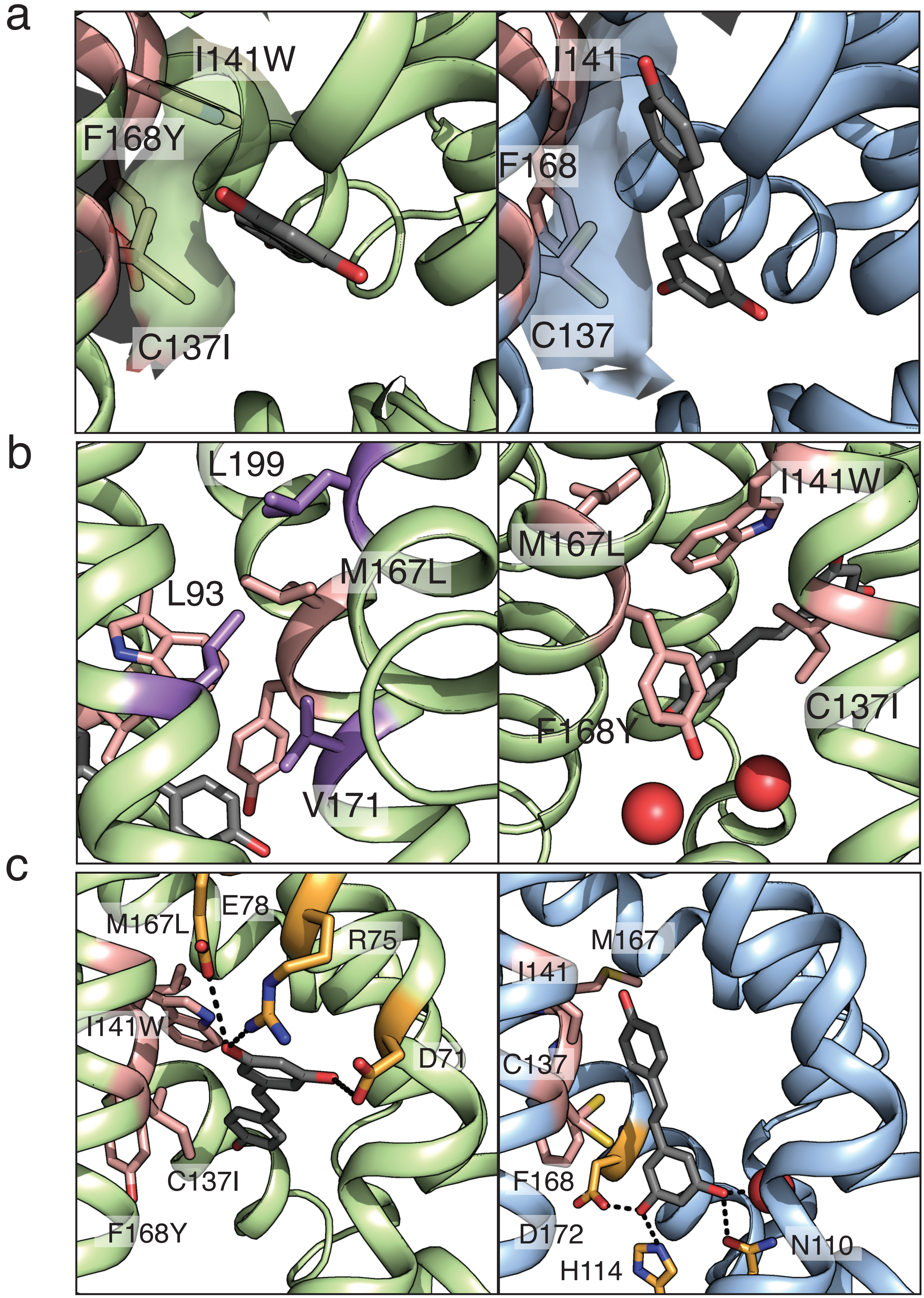

Supplementary Figure 13: Interactions of mutated positions and alternate hydrogen bonding networks

(a) The C137I mutation creates a small cleft in the binding pocket that can enhance shape complementarity to resveratrol in the horizontal binding mode. The quadruple mutant (left) is shown in comparison to wildtype (right). The van der Waals surface of residue 137 and 141 is shown for both structures. Positions 137, 141, 167, and 168 are shown as pink sticks. **(b)** (Left) M167L creates nonpolar interactions with residues in helices composing the binding pocket and dimerization interface (purple sticks). Mutated positions 137, 141, 167, and 168 are shown in pink. 167 also plays a role in positioning the I141W side chain. (Right) The F168Y substitution enables the formation of additional hydrogen bonds to solvent that can create a hydrogen bond network with D172. Water molecules are shown as red spheres. **(c)** The hydrogen bond network differs for the quadruple mutant between chain A and chain B due to the slightly different position of the resveratrol molecules in each. (Left) In the quadruple mutant, D71, R75 and E78 (orange) make hydrogen bonds with the resveratrol molecules. (Right) The hydrogen bonding network of wildtype chain A is identical to chain B. Water molecules are shown as red spheres.

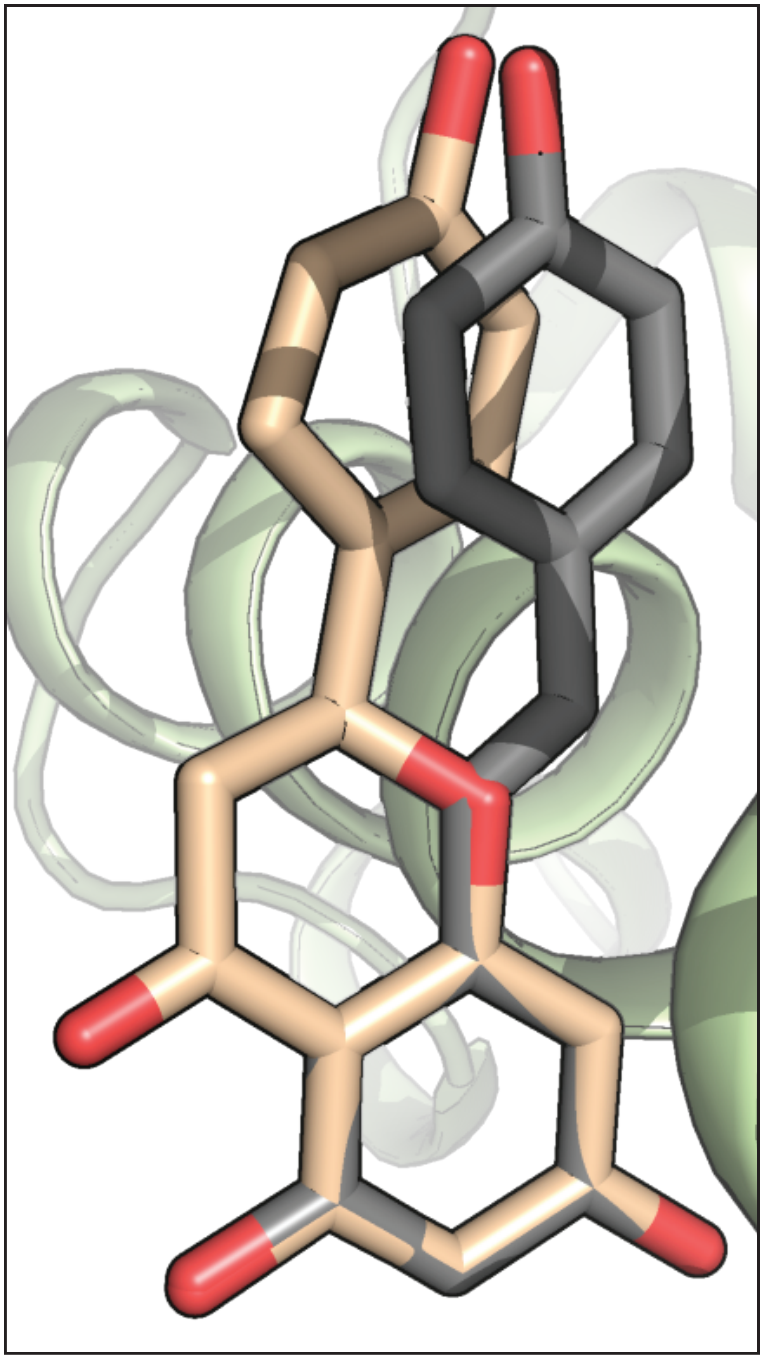

Supplementary Figure 14: Naringenin and resveratrol overlap
Naringenin (in brown) derived from a previous structure (PDB: 2UXU) is overlapped with resveratrol (in grey) via the pair_fit function in Pymol. The structures of each ligand are similar with respect to the location of hydroxyl groups, but differ by the addition of a carbonyl in the 4-chromanone backbone of naringenin.

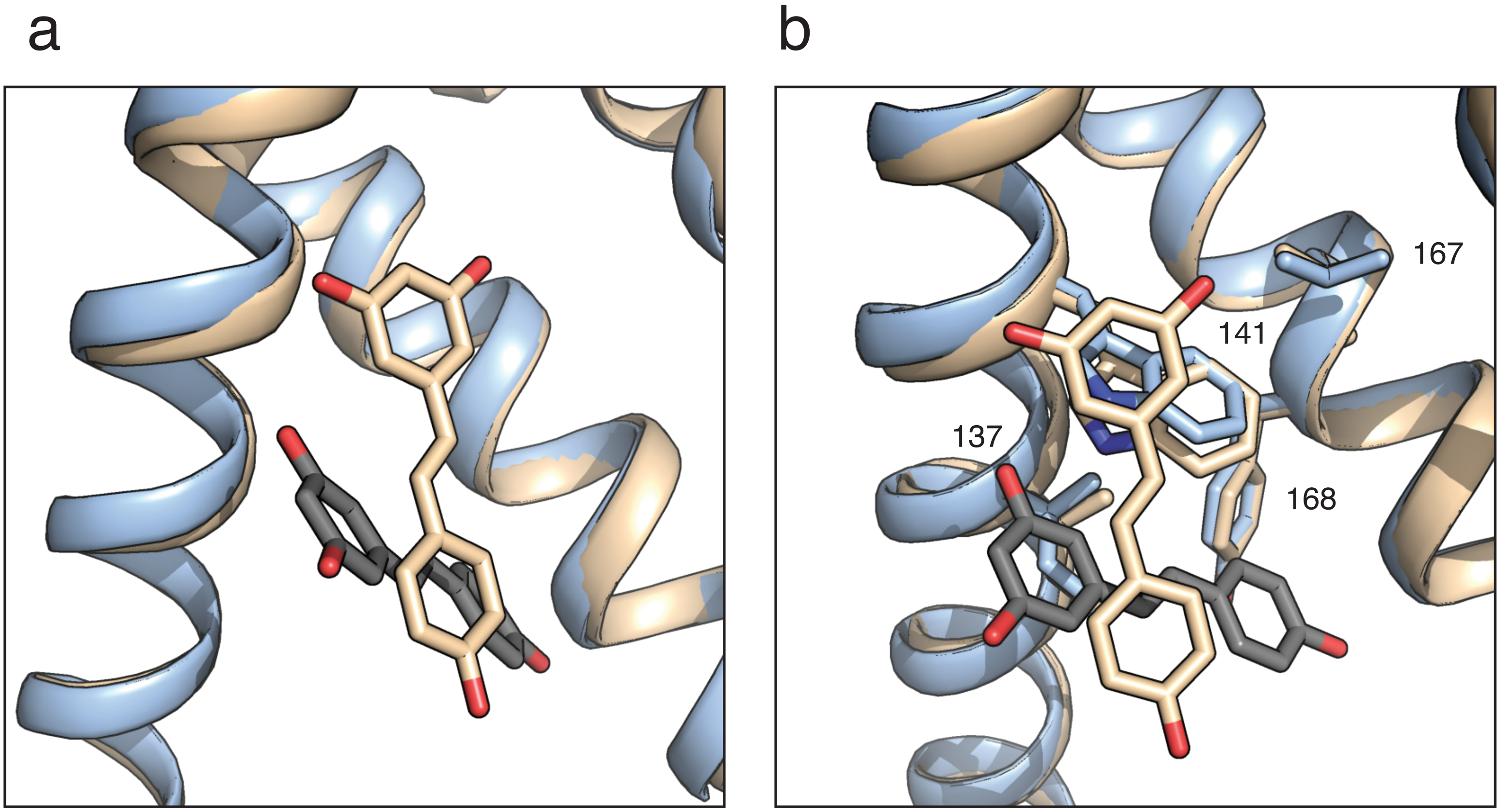

Supplementary Figure 15: Designed TtgR quadruple mutant binding pocket compared to crystal structure of TtgR quadruple mutant
The designed TtgR quadruple mutant (brown) is aligned to the crystal structure of the TtgR quadruple mutant (blue). (a) Resveratrol positions of the designed TtgR (brown) and the crystal structure (grey). (b) Mutated positions are shown as sticks. The design was unable to model the resveratrol in the horizontal orientation even though the residue rotamer states are similar to those in the crystal structure.

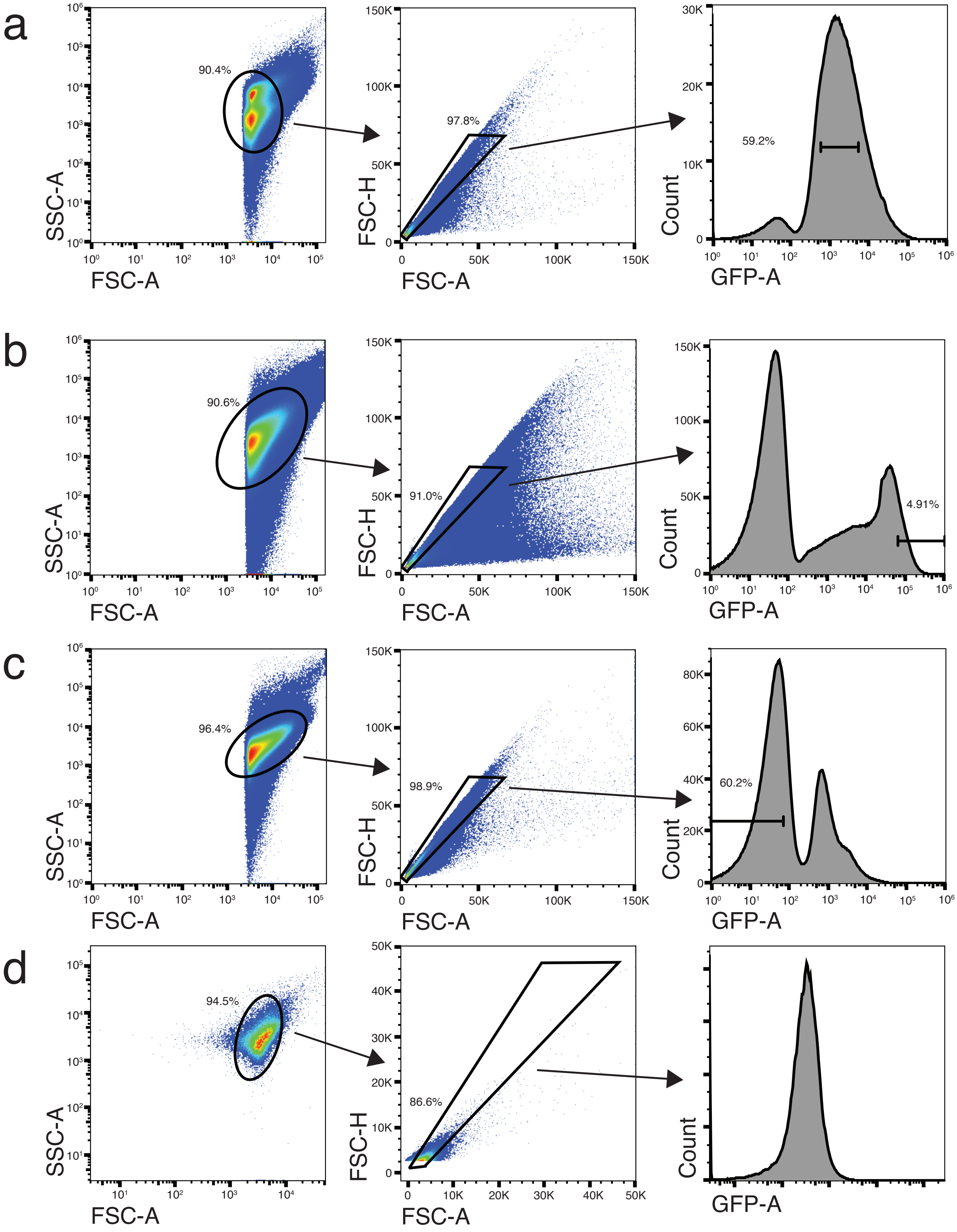

Supplementary Figure 16: Gating scheme for fluorescence-activated cell sorting and flow cytometry
(a) Gating strategy to sort cells containing a TtgR variant that is able to repress *sfGFP* expression. **(b)** Gating strategy to sort cells containing a TtgR variant that is able to induce *sfGFP* expression when exposed to 95.5µM resveratrol. **(c)** Gating strategy to isolate cells containing a TtgR variant that is able to both repress *sfGFP* expression in the absence of resveratrol and induce *sfGFP* expression in the presence of resveratrol. The sorting gating schemes are presented in Figure 1 and Supplementary Fig. 3. **(d)** Gating strategy to calculate fluorescence for flow cytometry experiments presented in Fig. 2, Fig. 3, Supplementary Fig. 4, Supplementary Fig. 6, Supplementary Fig. 7, Supplementary Fig. 8, Supplementary Fig. 9, Supplementary Fig. 16, and Supplementary Fig. 17.

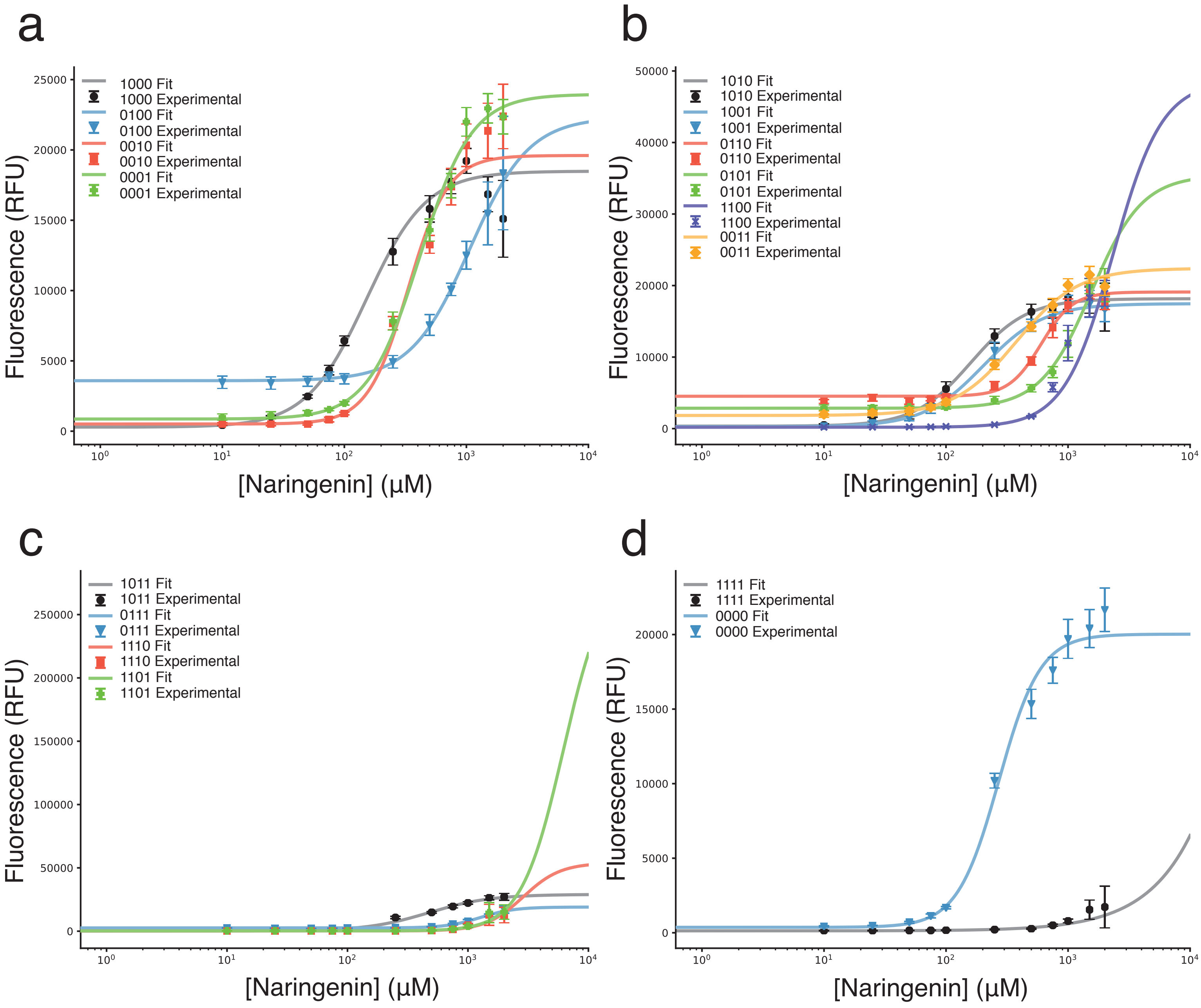

Supplementary Figure 17: Naringenin dose response curves
Dose response curves to naringenin for all 16 mutational combinations. Naringenin concentration varied between 0μM and 2000μM naringenin. Fit is shown as a solid line and experimental data is shown as markers with error bars. The marker denotes the averages and the error bars show the standard deviations of biological triplicate measurements (n=3) unless otherwise specified (see Methods). **(a)** Single mutant fits (1000, 0100, 0010, and 0001). **(b)** Double mutant fits (1010, 1001, 0110, 1100, and 0011). **(c)** Triple mutant fits (1011, 0111, 1110, and 1101). **(d)** Wildtype (0000) and quadruple mutant (1111) dose response curves.

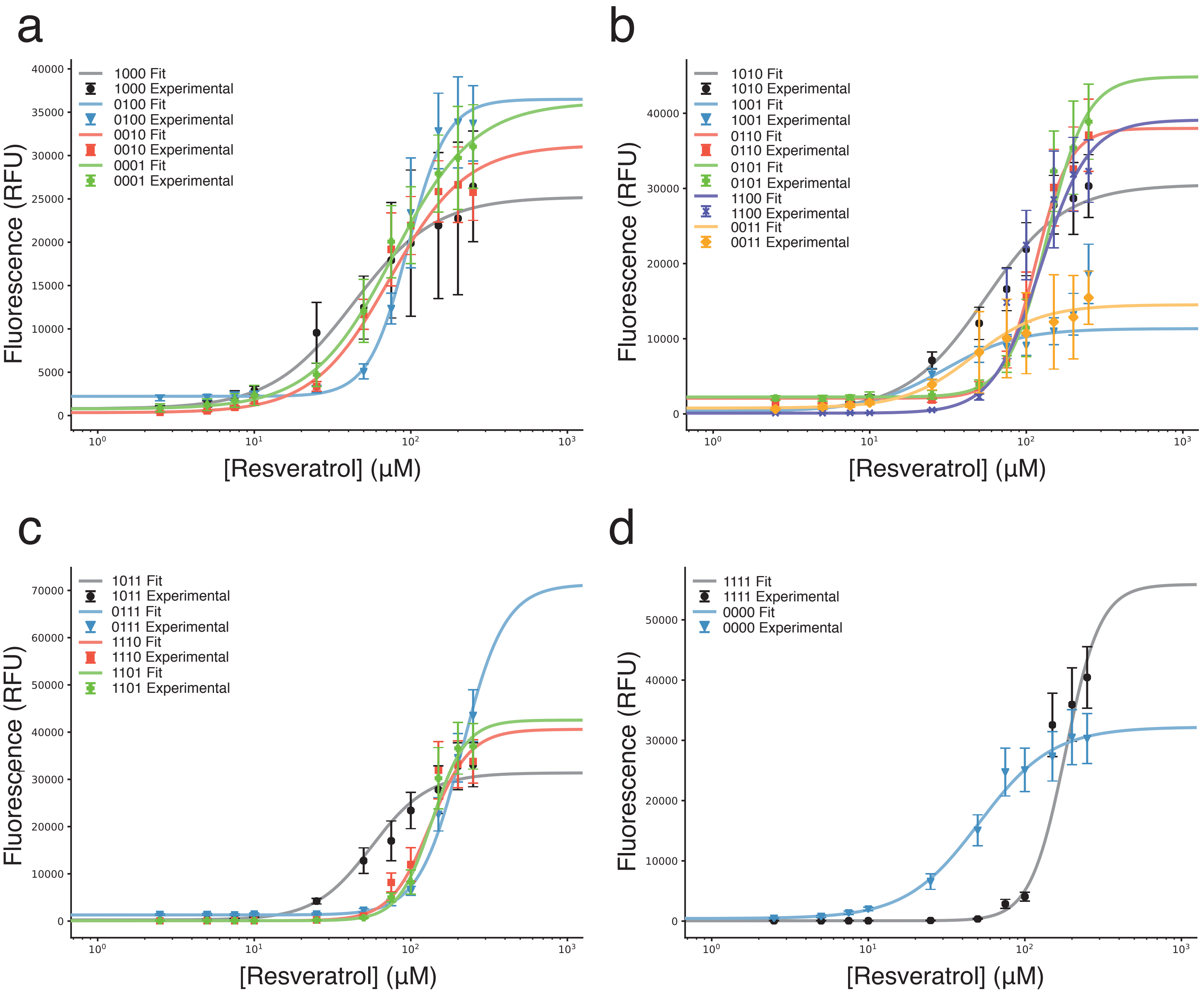

Supplementary Figure 18: Resveratrol dose response curves
Dose response curves for all 16 mutational combinations to resveratrol. Resveratrol concentration varied between 0μM and 250μM resveratrol. Fit is shown as a solid line and experimental data is shown as markers with error bars. The marker denotes the averages and the error bars show the standard deviations of biological triplicate measurements (n=3) unless otherwise specified (see Methods). **(a)** Single mutant fits (1000, 0100, 0010, and 0001). **(b)** Double mutant fits (1010, 1001, 0110, 1100, and 0011). **(c)** Triple mutant fits (1011, 0111, 1110, and 1101). **(d)** Wildtype (0000) and quadruple mutant (1111) dose response curves.

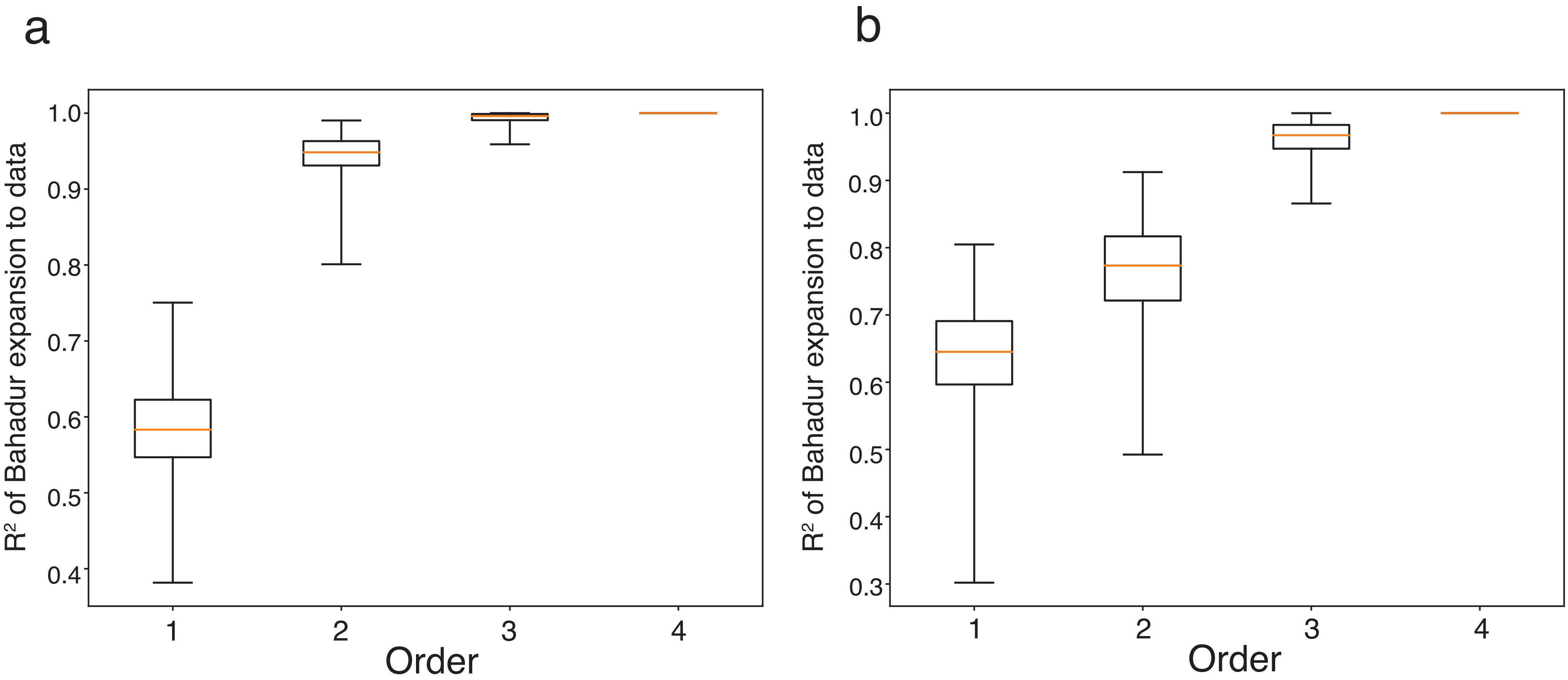

Supplementary Figure 19: Distribution of R^2 for first and higher order interactions for the full network

Distribution of R^2 values from the Bahadur expansion model applied to the full 16-member network after stochastic sampling (N=500) of fold induction values based on experimental averages and standard deviations. Boxplots of first, second, third, and fourth order interactions are shown for **(a)** resveratrol and **(b)** naringenin. The orange line is the median R^2 value for the distribution and the box encloses the interquartile range (IQR). The whiskers extend to the maximum and minimum R^2 values. The raw R^2 values for the fold induction, baseline fluorescence, EC_{50}, and can be found online at https://github.com/raman-lab/epistasis.

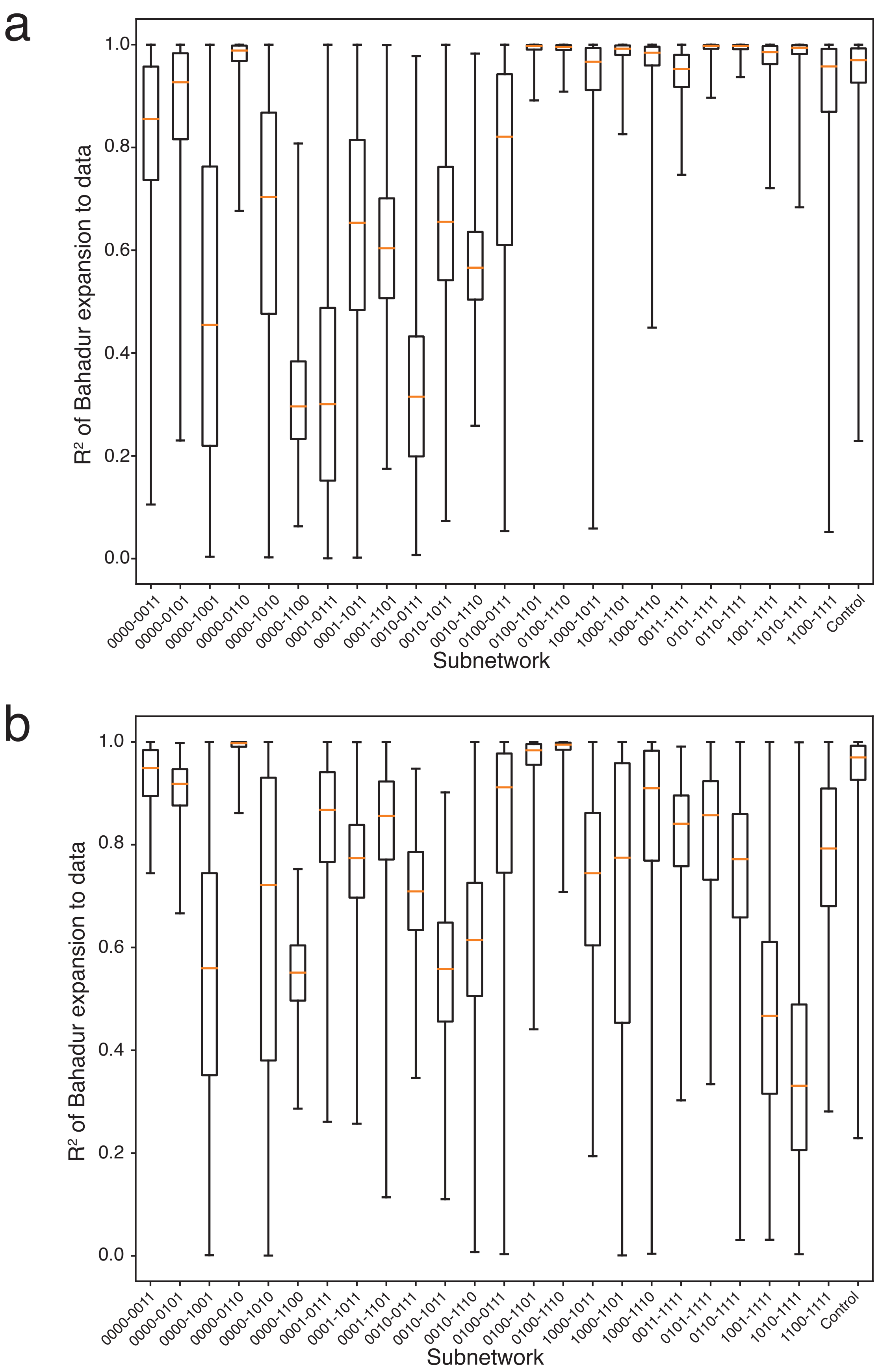

Supplementary Figure 20: Distribution of R^2 for first order interactions for individual subnetworks

Distribution of R^2 values from the Bahadur expansion model applied to each subnetwork after stochastic sampling of experimental fold induction values for **(a)** resveratrol or **(b)** naringenin based on experimental averages and standard deviations. Each subnetwork network was modeled 500 times by stochastic sampling. The orange line is the median R^2 value for the distribution and the box encloses the interquartile range (IQR). The whiskers extend to the maximum and minimum R^2 values.The raw R^2 values for the fold induction, baseline fluorescence, EC_{50}, and can be found online at https://github.com/raman-lab/epistasis.

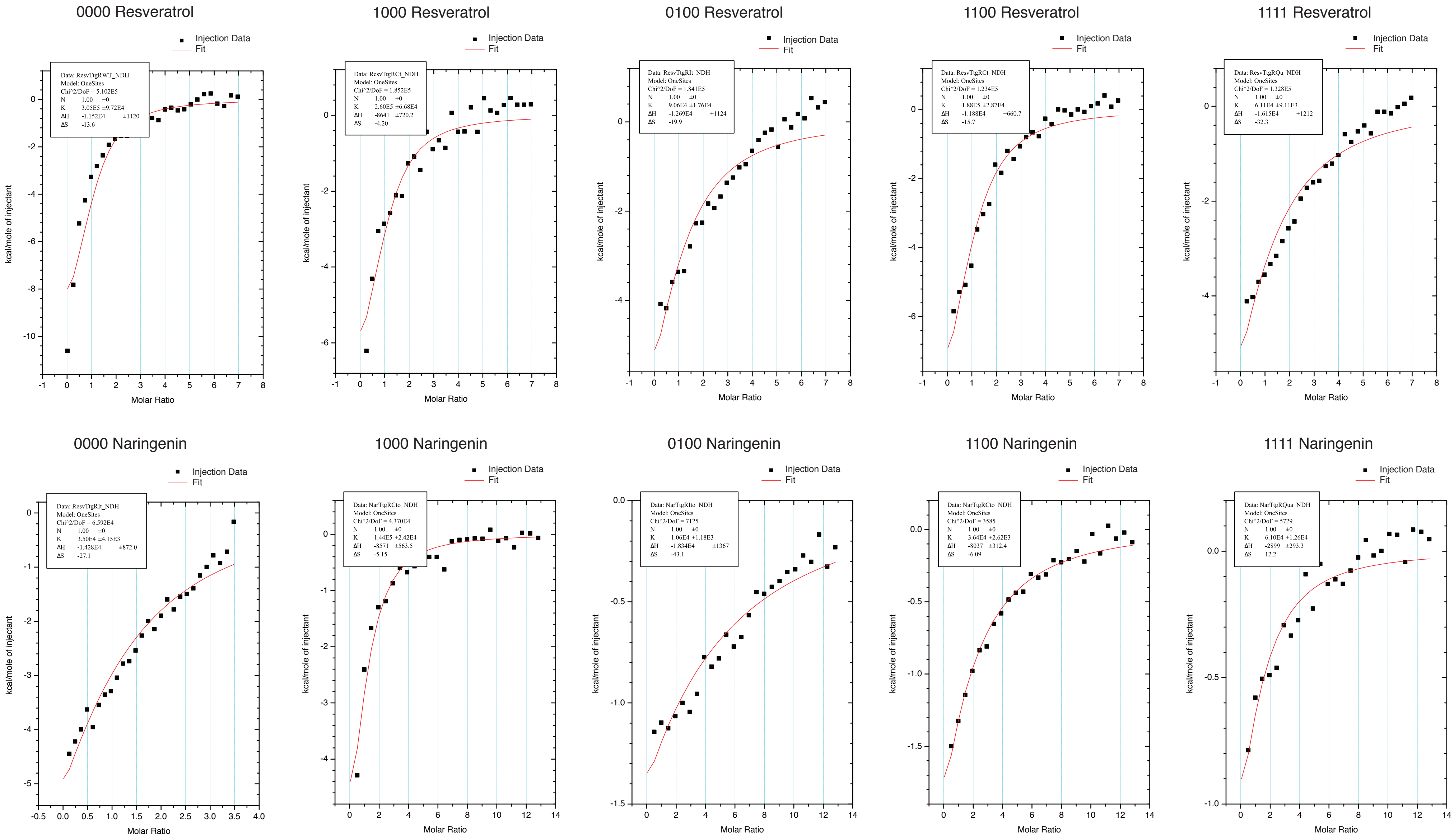

Supplementary Figure 21: Estimating binding parameters from isothermal calorimetry of wildtype TtgR and variants Isothermal titration calorimetry experimental data for affinity of TtgR mutants to either naringenin or resveratrol. Heat per mole of ligand injected (kCal/mol) is plotted as a function of the molar ratio of ligand:protein. Binding parameters are estimated from single site binding model fits using Origin 7.0 software (MicroCal). Due to low affinities for both naringenin and resveratrol, stoichiometry was fixed to 1 for both naringenin and resveratrol (see methods).

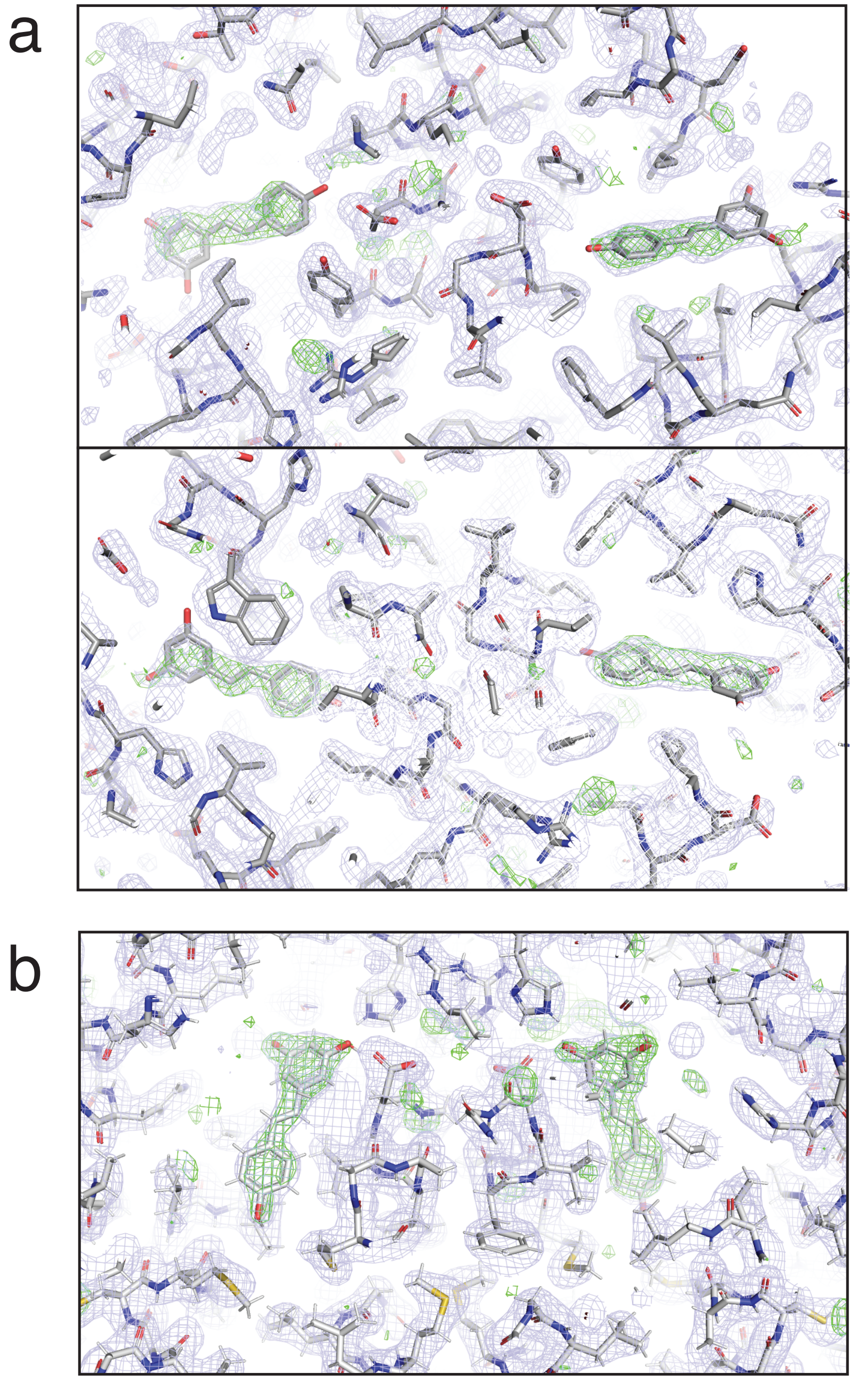

Supplementary Figure 22: mF_o-DF_c and $2mF_o$-DF_c omit maps for resveratrol-bound quadruple mutant TtgR and resveratrol-bound wildtype TtgR
Maps for the protein density were calculated in phenix from deposited models and structure factor amplitudes. The mF_o-Df_c map is contoured at 3σ and the $2mF_o$-DF_c maps were contoured at 2σ. The mF_o-DF_c omit map is shown as green wires while the $2mF_o$-DF_c omit map is shown in grey. **(a)** mF_o-DF_c and $2mF_o$-DF_c omit maps for resveratrol-bound quadruple mutant TtgR. Chain A is shown in the top panel on the left and chain B is on the right. The lower panel depicts chain C (left) and chain D (right). **(b)** mF_o-DF_c and $2mF_o$-DF_c omit maps for resveratrol-bound wildtype TtgR. Chain A is shown on the left and chain B is shown on the right.

Supplementary Tables

Name	Sequence
KN_E1	TATCACGAGGCCCTTTCGTCTTCACCACCCAGCAGTATTGACAAACAAC
KN_E2	TTCATGGTTGTTTGTCAATACTGCTGGGTGggcgcgccatgactaagcttttcattgtct
KN_E3	aaagttaaatgTTGCTAAGGATTATACTTACATTCATGGTTGTTTGTCAATACTGCTGGG
KN_E4	atgtatatctccttcttaaagttaaatgTTGCTAAGGATTATACTTA
KN_E5	cagctcttcgcctttacgcatatgtatatctccttcttaaagttaaatgTT
KN_E6	GTGAAGACGAAAGGGCCTCG
KN_E7	atgcgtaaaggcgaagagctg
KN_E8	catgctgcttcatGtggtcc
KN_E9	GCTGGCAATTCCGACGTC
KN_E10	TTGACAATTAATCATCCGGC
KN_E11	CGAGCCGGATGATTAATTGTCAA
KN_E12	TGAattagcagaaagtcaaaagcctccga
KN_E13	tcggaggcttttgactttctgctaatTCATTATTTGCGCAGCGCCGG
KN_E14	gCGATCGTGCCCACCT
KN_E15	GTGCGGGCTCCAACT
KN_E16	ggCTGGTGCGTCGTCT
KN_E17	cGGGAAGTGTTCGCCG
KN_E18	GGTCTCGGTTCTGGATGCACGTACCCGTCGC
KN_E19	GGTCTCGCAGTGCCTGAACCAGTTCGGC
KN_E20	GGTCTCGCGTTGGCTGCTGCTGCCGGATAG
KN_E21	GGTCTCGATCCAGCACGGCGCTCTGGCGC
KN_E22	GAAATTCGTCAGCAGCGCCAGAGCGCCGTGCTGGATattCATAAAGGTATCACC
KN_E23	GAAATTCGTCAGCAGCGCCAGAGCGCCGTGCTGGATTGTCATAAAGGTtggACC
KN_E24	GAAATTCGTCAGCAGCGCCAGAGCGCCGTGCTGGATattCATAAAGGTtggACC
KN_E25	GAAATTCGTCAGCAGCGCCAGAGCGCCGTGCTGGATTGTCATAAAGGTATCACC
KN_E26	CAGCAGCAGCCAACGGCGAATCAGGCCATCCACATAGGCAAAcagCGCAACCGC
KN_E27	CAGCAGCAGCCAACGGCGAATCAGGCCATCCACATAGGCataCATCGCAACCGC
KN_E28	CAGCAGCAGCCAACGGCGAATCAGGCCATCCACATAGGCatacagCGCAACCGC
KN_E29	CAGCAGCAGCCAACGGCGAATCAGGCCATCCACATAGGCAAACATCGCAACCGC
KN_E30	TTTTGTTTAACTTTAAGAAGGAGATATACATATGaaaatcgaagaaggtaaactggtaat
KN_E31	CATATGTATATCTCCTTCTTAAAGTTAAACAAAA
KN_E32	attgaaaatataaattttcGTGGTGGTGGTGGTGGtcgccgttaattaaagtctgcg
KN_E33	CCACCACCACgaaaatttatattttcaatctATGGTGCGTCGCACCAAAGAAGAAG
KN_E34	CTTTGTTAGCAGCCGGATCTCATTATTTGCGCAGCGCCGGGCTCAG
KN_E35	TGAGATCCGGCTGCTAACAAAGCCCGAAAGGA

Supplementary Table 1: Primers
Names and sequences of all primers used in this study.

Binary	Average	St. Dev
00	3.1	0.93
01	24.5	7.35
10	56.1	16.83
11	74.4	22.32

Supplementary Table 2: Control additive data set

A set of random values that are additive with respect to the mean. The standard deviation of each datapoint is 30% of the mean. This control set was used to calculate the R^2 for comparison to the subnetwork Bahadur expansions.

	Quadruple Mutant (Apo) 7K1A	TtgR QM STL 7KD8	wtTtgR STL 7K1C
**Wavelength**	0.9786	1.078	0.9786
**Resolution range**	32.14 - 1.75 (1.813 - 1.75)	25.65 - 1.71 (1.771 - 1.71)	28.63 - 1.9 (1.968 - 1.9)
**Space group**	C 2 2 21	P 1	C 2 2 21
**Unit cell**	57.92 64.28 223.87 90 90 90	43.497 43.587 115.942 97.969 98.648 96.761	57.73 64.5 223.22 90 90 90
**Total reflections**	578957 (41577)	311123 (31645)	481016 (49244)
**Unique reflections**	42585 (4137)	82029 (8133)	33367 (3316)
**Multiplicity**	13.6 (10.1)	3.8 (3.9)	14.4 (14.9)
**Completeness (%)**	99.71 (97.73)	91.78 (91.33)	99.88 (99.97)
**Mean I/sigma(I)**	16.20 (1.29)	13.88 (1.92)	22.92 (2.06)
**Wilson B-factor**	35.18	31.18	36.4
**R-merge**	0.09432 (1.303)	0.04573 (0.5776)	0.07704 (1.294)
**R-meas**	0.09805 (1.373)	0.05349 (0.6697)	0.07997 (1.339)
**R-pim**	0.02647 (0.4257)	0.02758 (0.3382)	0.02123 (0.3446)
**CC1/2**	0.987 (0.578)	0.998 (0.901)	0.999 (0.739)
**CC***	0.997 (0.856)	1 (0.974)	1 (0.922)
**Reflections used in refinement**	42566 (4136)	81923 (8104)	33349 (3315)
**Reflections used for R-free**	1979 (197)	1975 (197)	2020 (202)
**R-work**	0.1966 (0.3962)	0.1927 (0.2891)	0.1833 (0.3081)
**R-free**	0.2400 (0.3854)	0.2406 (0.3556)	0.2246 (0.3837)
**CC(work)**	0.955 (0.728)	0.959 (0.912)	0.963 (0.810)
**CC(free)**	0.920 (0.647)	0.930 (0.871)	0.948 (0.650)
**Number of non-hydrogen atoms**	3609	7247	3552
**macromolecules**	3321	6841	3322
**ligands**	2	72	36
**solvent**	286	334	194
**Protein residues**	413	830	415
**RMS(bonds)**	0.003	0.006	0.016
**RMS(angles)**	0.48	0.72	1.23
**Ramachandran favored (%)**	99.27	99.27	99.51
**Ramachandran allowed (%)**	0.73	0.61	0.49
**Ramachandran outliers (%)**	0	0.12	0
**Rotamer outliers (%)**	0.58	0.84	0.86
**Clashscore**	2.11	2.17	2.53
**Average B-factor**	39.41	40.02	42.83
**macromolecules**	39.27	39.83	42.66
**ligands**	35.92	53	52.81
**solvent**	41.05	41.07	43.92
**Number of TLS groups**	14	22	1

Supplementary Table 3: Crystallography refinement statistics

Refinement statistics for three structures: apo quadruple mutant TtgR (7K1A), wildtype TtgR bound to resveratrol (7K1C), and quadruple mutant TtgR bound to resveratrol (7KD8). Statistics for the highest resolution shell are shown in parentheses.

	0000	0001	0010	0011	0100	0101	0110	0111	1000	1001	1010	1011	1100	1101	1110	1111	Residue Interactions
Ψ_0	1	1	1	1	1	1	1	1	1	1	1	1	1	1	1	1	0
Ψ_1	-1	-1	-1	-1	-1	-1	-1	-1	1	1	1	1	1	1	1	1	1
Ψ_2	-1	-1	-1	-1	1	1	1	1	-1	-1	-1	-1	1	1	1	1	2
Ψ_3	-1	-1	1	1	-1	-1	1	1	-1	-1	1	1	-1	-1	1	1	3
Ψ_4	-1	1	-1	1	-1	1	-1	1	-1	1	-1	1	-1	1	-1	1	4
Ψ_5	1	1	1	1	-1	-1	-1	-1	-1	-1	-1	-1	1	1	1	1	1-2
Ψ_6	1	1	-1	-1	1	1	-1	-1	-1	-1	1	1	-1	-1	1	1	1-3
Ψ_7	1	-1	1	-1	1	-1	1	-1	-1	1	-1	1	-1	1	-1	1	1-4
Ψ_8	1	1	-1	-1	-1	-1	1	1	1	1	-1	-1	-1	-1	1	1	2-3
Ψ_9	1	-1	1	-1	-1	1	-1	1	1	-1	1	-1	-1	1	-1	1	2-4
Ψ_{10}	1	-1	-1	1	1	-1	-1	1	1	-1	-1	1	1	-1	-1	1	3-4
Ψ_{11}	-1	-1	1	1	1	1	-1	-1	1	1	-1	-1	-1	-1	1	1	1-2-3
Ψ_{12}	-1	1	-1	1	1	-1	1	-1	1	-1	1	-1	-1	1	-1	1	1-2-4
Ψ_{13}	-1	1	1	-1	-1	1	1	-1	1	-1	-1	1	1	-1	-1	1	1-3-4
Ψ_{14}	-1	1	1	-1	1	-1	-1	1	-1	1	1	-1	1	-1	-1	1	2-3-4
Ψ_{15}	1	-1	-1	1	-1	1	1	-1	-1	1	1	-1	1	-1	-1	1	1-2-3-4

Supplementary Table 4: Psi values for Bahadur Expansion

Psi values for all orders of interactions (right column) for each mutant.
