# Supplementary material for: Epistasis shapes the fitness landscape of an allosteric specificity switch
Source: Nat Commun. 2021 Sep 21;12:5562. doi: 10.1038/s41467-021-25826-7 (PMC8455584; doi:10.1038/s41467-021-25826-7)
Supplement: Supplementary file 2 — Reporting summary [file 41467_2021_25826_MOESM2_ESM.pdf]

nature research

Corresponding author(s): Srivatsan Raman

Last updated by author(s): Aug 11, 2021

Reporting Summary

Nature Research wishes to improve the reproducibility of the work that we publish. This form provides structure for consistency and transparency in reporting. For further information on Nature Research policies, see our Editorial Policies and the Editorial Policy Checklist.

Statistics

For all statistical analyses, confirm that the following items are present in the figure legend, table legend, main text, or Methods section.

n/a	Confirmed	
☐	☒	The exact sample size (*n*) for each experimental group/condition, given as a discrete number and unit of measurement
☐	☒	A statement on whether measurements were taken from distinct samples or whether the same sample was measured repeatedly
☒	☐	The statistical test(s) used AND whether they are one- or two-sided<br>*Only common tests should be described solely by name; describe more complex techniques in the Methods section.*
☒	☐	A description of all covariates tested
☒	☐	A description of any assumptions or corrections, such as tests of normality and adjustment for multiple comparisons
☐	☒	A full description of the statistical parameters including central tendency (e.g. means) or other basic estimates (e.g. regression coefficient) AND variation (e.g. standard deviation) or associated estimates of uncertainty (e.g. confidence intervals)
☒	☐	For null hypothesis testing, the test statistic (e.g. *F*, *t*, *r*) with confidence intervals, effect sizes, degrees of freedom and *P* value noted<br>*Give P values as exact values whenever suitable.*
☐	☒	For Bayesian analysis, information on the choice of priors and Markov chain Monte Carlo settings
☒	☐	For hierarchical and complex designs, identification of the appropriate level for tests and full reporting of outcomes
☒	☐	Estimates of effect sizes (e.g. Cohen's *d*, Pearson's *r*), indicating how they were calculated

Our web collection on statistics for biologists contains articles on many of the points above.

Software and code

Policy information about availability of computer code

Data collection	ITC data was collected from a MicroCal VP-ITC Microcalorimeter using VPViewer 2000 and Origin 7.0. Flow cytometry data was collected from a BD LSRFortessa using BD FACSDiva V8.0.
Data analysis	Flow cytometry data was analyzed in FlowJo V10. ITC data was analyzed using Origin 7.0. Further analysis and statistical calculations were made using Python 2.7 using custom scripts, which can be found at: https://github.com/raman-lab/epistasis. X-ray crystallography data was reduced using XDS (VERSION Mar 15, 2019 BUILT=20190315) and scaled with XSCALE (VERSION Mar 15, 2019 BUILT=20190315). Structures were solved by molecular replacement with Phaser V2.8.2 within the Phenix suite of programs V1.18.2_3874, automatically rebuilt with phenix.autobuild, iteratively improved with alternating rounds of rebuilding in Coot V0.9.5 and refinement using phenix.refine, and validated using MOLPROBITY V4.02-528. Pocket volume was calculated using POVME V3.0.

For manuscripts utilizing custom algorithms or software that are central to the research but not yet described in published literature, software must be made available to editors and reviewers. We strongly encourage code deposition in a community repository (e.g. GitHub). See the Nature Research guidelines for submitting code & software for further information.

Data

Policy information about availability of data

All manuscripts must include a data availability statement. This statement should provide the following information, where applicable:

- Accession codes, unique identifiers, or web links for publicly available datasets
- A list of figures that have associated raw data
- A description of any restrictions on data availability

All data generated prior or during analysis for the current study can be provided by the corresponding author upon reasonable request. All figures and supplementary figures have associated raw data. Crystal structures of the TtgR variants can be found in the Protein Data Bank (PDB ID: 7K1A, 7K1C, and 7KD8).

There are no restrictions on data availability.

Field-specific reporting

Please select the one below that is the best fit for your research. If you are not sure, read the appropriate sections before making your selection.

☒ Life sciences ☐ Behavioural & social sciences ☐ Ecological, evolutionary & environmental sciences

For a reference copy of the document with all sections, see nature.com/documents/nr-reporting-summary-flat.pdf

Life sciences study design

All studies must disclose on these points even when the disclosure is negative.

Sample size	Dose response curves used biological triplicate (N = 3), which is common with transcription factor-based assays in other systems (Schwarz, K., et al. Rewiring human cellular input–output using modular extracellular sensors. Nat Chem Biol 13, 202-209 (2017). https://doi.org/10.1038/nchembio.2253). Three replicates have also been used to analyze epistasis in a fitness landscape (Yang, G., Anderson, D.W., Baier, F. et al. Higher-order epistasis shapes the fitness landscape of a xenobiotic-degrading enzyme. Nat Chem Biol 15, 1120–1128 (2019). https://doi.org/10.1038/s41589-019-0386-3). Variants with high biological noise were assayed with an additional three replicates (N=6).
Data exclusions	No data was excluded
Replication	The biological replicates (N=3) used for the flow cytometry experiments showed reproducibility. All data can be found in the source data file. ITC experiments were not repeated.
Randomization	Randomization was not necessary because samples were separated based on TtgR variant encoded in a plasmid.
Blinding	Flow cytometry data acquisition and analysis is not affected by investigator bias that can be mitigated through blinding. ITC fits use an error-minimization algorithm to determine affinity and are not subject to investigator biases. Fitting the Hill equation to the dose response curves used a least squares method of fitting and was not subject to investigator bias. The structure was solved by individuals with no knowledge of the functional properties of the proteins.

Reporting for specific materials, systems and methods

We require information from authors about some types of materials, experimental systems and methods used in many studies. Here, indicate whether each material, system or method listed is relevant to your study. If you are not sure if a list item applies to your research, read the appropriate section before selecting a response.

Materials & experimental systems

n/a	Involved in the study
☒	☐ Antibodies
☒	☐ Eukaryotic cell lines
☒	☐ Palaeontology and archaeology
☒	☐ Animals and other organisms
☒	☐ Human research participants
☒	☐ Clinical data
☒	☐ Dual use research of concern

Methods

n/a	Involved in the study
☒	☐ ChIP-seq
☐	☒ Flow cytometry
☒	☐ MRI-based neuroimaging

Flow Cytometry

Plots

Confirm that:

☒ The axis labels state the marker and fluorochrome used (e.g. CD4-FITC).

☒ The axis scales are clearly visible. Include numbers along axes only for bottom left plot of group (a 'group' is an analysis of identical markers).

☒ All plots are contour plots with outliers or pseudocolor plots.

☒ A numerical value for number of cells or percentage (with statistics) is provided.

Methodology

Sample preparation	Colonies were selected and inoculated into 150uL LB in a 96-well plate. These grew in a microplate shaker to saturation (approximately 8 hours) at 37°C. The cultures were diluted 15X into fresh LB-kanamycin/spectinomycin in a 96-well plate with varying concentrations of either naringenin (0µM, 10µM, 25µM, 50µM, 75µM, 100µM, 250µM, 500µM, 750µM, 1000µM,

	1500µM, 2000µM) or resveratrol (0µM, 2.5µM, 5µM, 7.5µM, 10µM, 25µM, 50µM, 75µM, 100µM, 150µM, 200µM, 250µM). A series of naringenin and resveratrol stock concentrations were made such that a 50X or a 100X dilution, respectively, would yield the desired concentrations in the assay. The assay was incubated in the microplate shaker for 14 hours at 37°C shaking. Cells were diluted 50X in ice cold PBS prior to flow cytometry.
Instrument	BD LSRFortessa X-20 Special Order System
Software	BD FACSDiva 8.0 was used to collect the data. Analysis was done in FlowJo v10 and using custom Python 2.7 scripts that can be found at: https://github.com/raman-lab/epistasis
Cell population abundance	No sorting was conducted
Gating strategy	A gate on FSC-A and SSC-A was drawn to capture E.coli. A second gate using FSC-A and FSC-H was drawn to capture singlets.

☒ Tick this box to confirm that a figure exemplifying the gating strategy is provided in the Supplementary Information.
